# Supramolecular reactions of metallo-architectures: Ag_2_-double-helicate/Zn_4_-grid, Pb_4_-grid/Zn_4_-grid interconversions, and Ag_2_-double-helicate fusion†
†Electronic supplementary information (ESI) available. See DOI: 10.1039/c5sc04403k


**DOI:** 10.1039/c5sc04403k

**Published:** 2016-02-29

**Authors:** Adrian-Mihail Stadler, Juan Ramírez, Jean-Marie Lehn, Bruno Vincent

**Affiliations:** a Université de Strasbourg , CNRS , UMR 7006 , ISIS , 8 Allée G. Monge , Strasbourg , France . Email: mstadler@unistra.fr; b Institute of Nanotechnology (INT) , Karlsruhe Institute of Technolgoy (KIT) , 76344 , Eggenstein-Leopoldshafen , Germany; c Institut Pasteur Paris , 28 Rue du Docteur Roux , 75015 Paris , France; d Service de RMN , Faculté de Chimie , 1 Rue B. Pascal , Strasbourg , France

## Abstract

Supramolecular reactions are of importance in many fields. We report herein three examples where complexes of hydrazone-based ligands are involved. A Ag_2_-double-helicate was converted, by treatment with Zn(OTf)_2_, into a Zn_4_-grid (exchange of metal ions and change of the nature of the initial complex). A Pb_4_-grid was converted, upon reaction with ZnCl_2_ or ZnBr_2_, into a Zn_4_-grid (exchange of metal ions, but conservation of the nature of the initial complex). The reverse conversions were also achieved. The fusion of a Ag_2_-double-helicate with another Ag_2_-double-helicate was performed (exchange of ligands, but conservation of the nature of the complexes) and resulted in a mixture of three helicates (two homostranded ones and one heterostranded one).

## Abstract 2

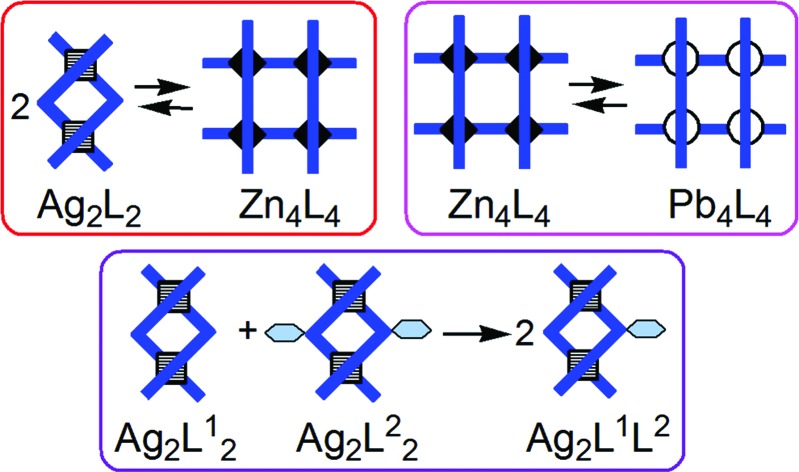
A Ag^+^ dinuclear double-helicate was converted into a Zn^2+^ tetranuclear grid, and a Pb^2+^ grid, into a Zn^2+^ grid; a Ag^+^ heterostranded double helicate was obtained from two homostranded helicates.

## Introduction

Like covalent molecules, supramolecular^1^ assemblies may participate in various reactions. The understanding of supramolecular reactions is of much interest because they are involved in many areas such as complex chemical systems and networks,^2^ adaptive^3^ and stimuli-responsive^4^ chemical systems, fabrication of nanodevices and materials,^4^ biomolecular processes. Thus, in the complexity and diversity of supramolecular chemistry, the reactivity of supramolecules plays a crucial role. It includes the processes:

(a) of (self)assembly (*i.e.* formation of supramolecular architectures through assembly, but also their participation, as subunits, in more complex assemblies), and correlatively, partial or total disassembly;

(b) of partial or total reorganization or exchange (at the supramolecular and, additionally and possibly, at the covalent level), that involves the breaking of several or all of the initial supramolecular connections and formation of new ones;

(c) without breaking or formation of new supramolecular connections (*e.g.* covalent modifications after self-assembly^5^).

Amongst supramolecular architectures, double helices and helicates,^6^ as well as grids^7^ arouse much interest and work. For example, DNA^8^ and the ion channel generated by gramicidine^9^ have a double helical structure, and there are double helical complexes that act as molecular machines^10^ or catalysts.^11^ Grid-like complexes have been studied for their electrochemical and magnetic properties,^7^ for their capacity to encapsulate ions^12^ or as starting materials for building more complex architectures (*e.g.* a Solomon link^13^), amongst other things. However, supramolecular interconversions of grids and helicates have not, except several examples,^14^,^15^ been much explored.

**Fig. 1:**
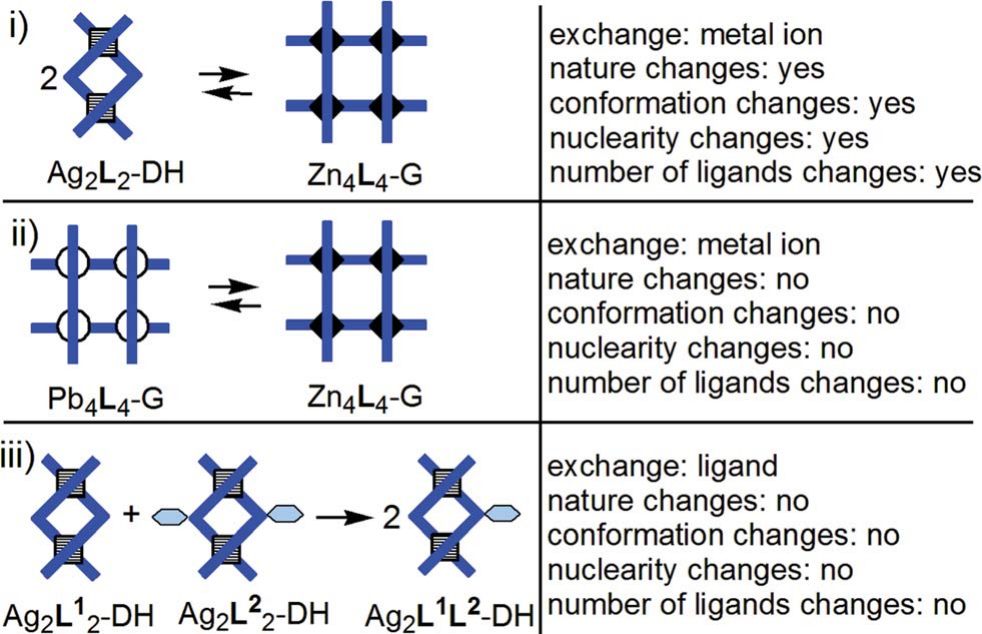
Stylized representation of the three types of supramolecular reactions reported herein.

**Fig. 2:**
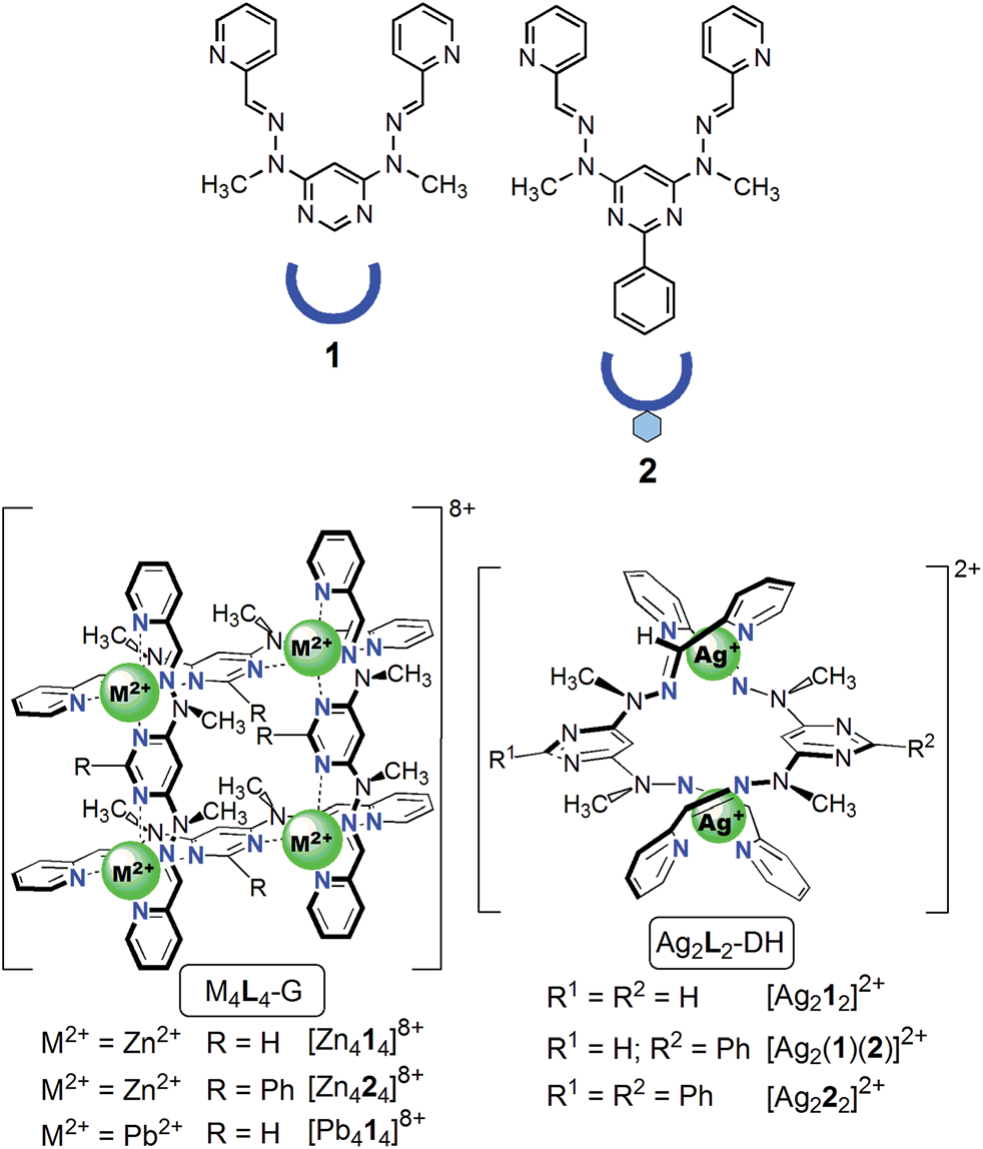
Structural formulae and stylized representations of ligands **1** and **2**, and of grids and double helicates.

**Fig. 3:**
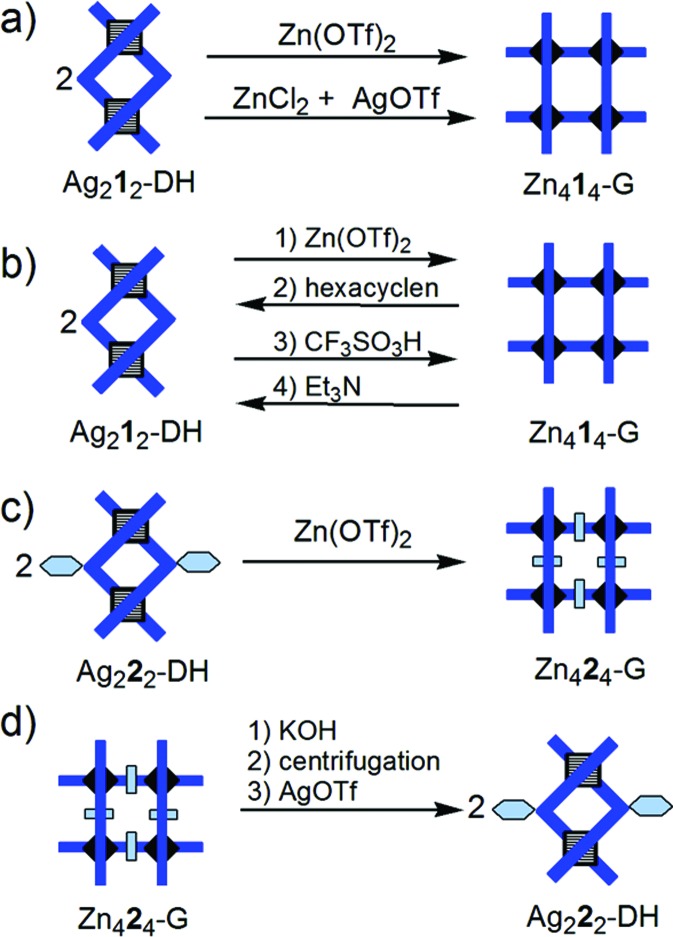
Stylized representation of: (a) the conversion of Ag_2_**1**_2_-DH double helicate into the Zn_4_**1**_4_-G grid; (b) the interconversion Ag_2_**1**_2_-DH/Zn_4_**1**_4_-G; (c) the conversion of Ag_2_**2**_2_-DH double helicate into the Zn_4_**2**_4_-G grid; (d) the conversion of Zn_4_**2**_4_-G grid into Ag_2_**2**_2_-DH double helicate. Charges and stoichiometric coefficients are omitted for simplicity.

**Fig. 4:**
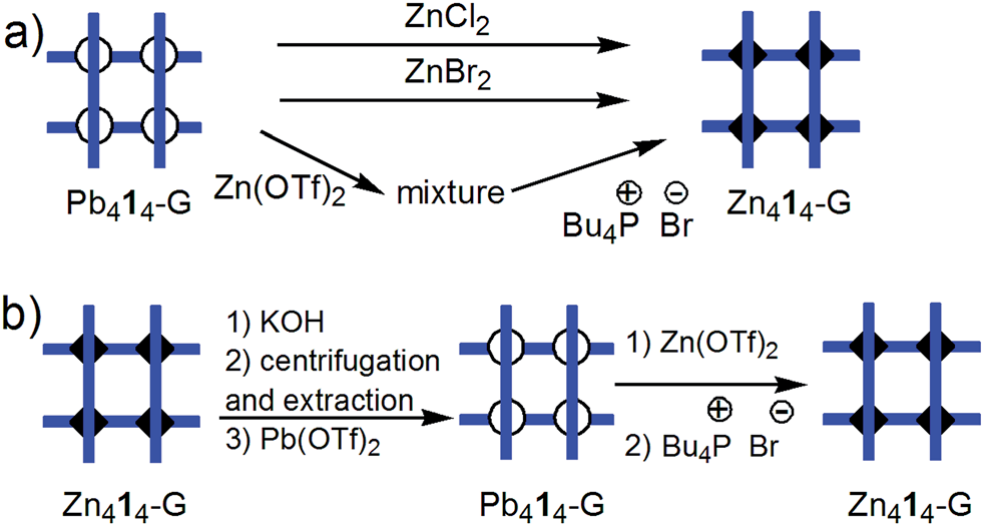
Stylized representation of: (a) reaction of Pb_4_**1**_4_-G grid with ZnCl_2_, ZnBr_2_, and Zn(OTf)_2_ and Bu_4_P^+^Br^–^ (solvent CD_3_CN); (b) Zn_4_**1**_4_-G/Pb_4_**1**_4_-G and Pb_4_**1**_4_-G/Zn_4_**1**_4_-G conversions. Charges and stoichiometric coefficients are omitted for simplicity.

**Fig. 5:**
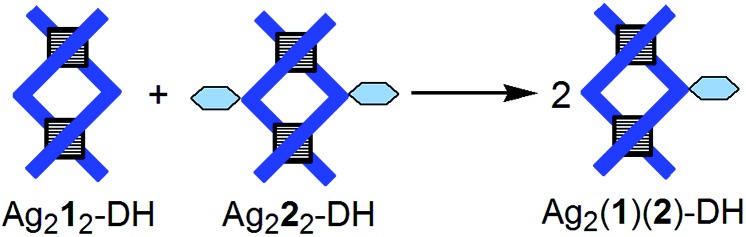
Stylized representation of the fusion (conproportionation) reaction of double helicates Ag_2_**1**_2_-DH and Ag_2_**2**_2_-DH with formation of heterostranded species Ag_2_(**1**)(**2**)-DH. Charges are omitted for simplicity.

With these ideas in mind – and using principles such as the displacement of an equilibrium through precipitation, and the preference of Ag^+^ for tetrahedral and of Zn^2+^ for octahedral coordination – we designed, as reported herein, three supramolecular reactions^16^ of reorganization and exchange (Fig. 1) involving grids and double helicates. They are related through the ligands^17^ (which are pyrimidine-bis-hydrazones;^18^Fig. 2) that produce the supramolecular complexes, as well as through the nature of complexes, and occur due to the dynamic character of the present metal–ligand connections. These reactions (Fig. 1) can be seen as:

(i) a change of the nature of the supramolecular architecture, from a Ag^+^ dinuclear double helicate (DH) into a Zn^2+^ tetranuclear grid (G), induced by replacement of Ag^+^ by Zn^2+^ (Fig. 3). In this reaction, not only the nature of the complex and that of the metal ion change, but also the conformation of the ligand (helical → unfolded), the charge (2^+^ → 8^+^) and the nuclearity of the complex (2 → 4) and the number of ligands per complex (2 → 4). In regard to this last change, this process can be compared with the conversion or the equilibrium between supramolecular dimer and tetramer of bioactive proteins,^19^ or between other homo-oligomers^20^ with influence on the protein functions.

(ii) a substitution^21^ (metal ion exchange or transmetallation), in a sole operation, of the four Pb^2+^ ions of a grid-like^22^ complex by Zn^2+^ ions (Fig. 4);

(iii) a fusion (conproportionation)^23^ between two Ag^+^ double helicates^24^ (Fig. 5).

While in case (ii) the equilibrium is shifted towards the Zn^2+^ grid through the precipitation of Pb^2+^ as its halides (chloride and bromide), in cases (i) and (iii), the conversions can be done without precipitation.

## Results and discussion

(i) The conversion Ag_2_**L**_2_-DH → Zn_4_**L**_4_-G (**L** = **1**, **2**) through transmetallation is a dramatic reorganization of the nature of the metallo-supramolecular architecture induced by the replacement of Ag^+^ by Zn^2+^ (Fig. 3a and c): 2 Ag_2_**L**_2_-DH + 4 Zn^2+^ → Zn_4_**L**_4_-G + 4 Ag^+^. Ag^+^ prefers a tetrahedral coordination geometry which is, in the case of ligands **1** and **2**, achieved from 2 two-Nsp^2^-atom bidentate pyridine-hydrazone sites. In this way, Ag^+^ induces the formation of double helicates with ligands **1** and **2**. Zn^2+^ prefers an octahedral coordination environment that results from **2** three-Nsp^2^-atom tridentate sites of type pyridine-hydrazone-pyrimidine, thus generating a grid.

Reaction of 1 equiv. of Ag_2_**L**_2_-DH15*c* with 2 equiv. of Zn(OTf)_2_ (OTf^–^ = CF_3_SO^3–^) produces – without the need to precipitate Ag^+^ as a halide – the corresponding grid Zn_4_**L**_4_-G15b,22a (solvent: CD_3_NO_2_ with 6–14% CD_3_CN; ESI, pp. S9–S11†). Where ZnCl_2_ is used in the reaction with Ag_2_**1**_2_-DH, two equivalents of AgOTf per equiv. of DH are required according to the equation (ESI p. S8†):2Ag_2_**1**_2_-DH + 4ZnCl_2_ + 4Ag^+^ → Zn_4_**1**_4_-G + 8AgCl

On treatment of the double helicate Ag_2_**2**_2_-DH in CD_3_NO_2_ with 2 equiv. of Zn(OTf)_2_ – added as a solution in a small volume of CD_3_CN, or as a solid – the grid Zn_4_**2**_4_-G was obtained. When the double helicate Ag_2_**1**_2_-DH in CD_3_NO_2_ was treated with 2 equiv. of Zn(OTf)_2_, added as a solution in a small volume of CD_3_CN (about 6–14% of the CD_3_NO_2_ volume), the grid Zn_4_**1**_4_-G was obtained. When Zn(OTf)_2_ was added as a solid, without CD_3_CN, was obtained a mixture without the Zn_4_**1**_4_-G grid; addition of a small volume of CH_3_CN (about 6–14% of the CD_3_NO_2_ volume) to this mixture produced the expected grid Zn_4_**1**_4_-G. A possible explanation could be that, in the case of the reaction Ag_2_**1**_2_-DH → Zn_4_**1**_4_-G, the CH_3_CN acts as a coordinating species for the Ag^+^ ions and so contributes to the displacement of the equilibrium from the double helix towards the grid. The grid Zn_4_**2**_4_-G should be – due to the π-stacking aromatic interaction between a phenyl ring and the two ligands between which that phenyl is located within the grid – more stable than the grid Zn_4_**1**_4_-G. This stability may be sufficient to make possible the formation of the grid Zn_4_**2**_4_-G from the corresponding double helicate without, unlike in the case of the grid Zn_4_**1**_4_-G, the assistance of CH_3_CN.

DOSY NMR was also used to study the conversion Ag_2_**L**_2_-DH → Zn_4_**L**_4_-G (**L** = **1**, **2**). As expected, the volume of the grid species obtained from double helicates on treatment with Zn(OTf)_2_ was found in agreement with that of the grid prepared from the free ligands **L** and Zn(OTf)_2_.

The reverse conversion Zn_4_**L**_4_-G→ Ag_2_**L**_2_-DH can be done as follows: after treatment of the grid with KOH, the solvent (CD_3_CN or CD_3_NO_2_) is removed, and the ligand is extracted with CDCl_3_ and separated from the solid residue (by centrifugation or filtration); after removal of CDCl_3_, CD_3_NO_2_ is added, then AgOTf is added to form the helicate. In order to simplify the procedure, we used ligand **2** and a mixture of CDCl_3_ and CD_3_NO_2_ where ligand **2**, as well as the corresponding grid and double helicate were soluble. After precipitation of Zn^2+^ with KOH, the mixture was centrifuged (the ligand **2** being soluble in the mixture of solvents), and to the recovered liquid phase AgOTf was added to produce the Ag_2_**2**_2_-DH (ESI, p. S13†).

In a pH-dependent system (Fig. 3b), the interconversion between Ag_2_**1**_2_-DH and Zn_4_**1**_4_-G was achieved as follows (ESI, p. S10†): the grid was generated from the double helicate by reaction with Zn^2+^; then, Zn^2+^ was complexed with hexacyclen, and the double helicate was regenerated; partial protonation of hexacyclen with TfOH caused release of Zn^2+^ and formation of the grid (incomplete yield); finally, addition of triethylamine reactivated the hexacyclen that again encapsulated Zn^2+^ and resulted in the reformation of the double helicate.

(ii) The Pb_4_**1**_4_-G → Zn_4_**1**_4_-G conversion (Fig. 4a) can formally be seen as a substitution of Pb^2+^ by Zn^2+^ ions, although the real mechanism, involving breaking and formation of supramolecular bonds, must be more complex. Reaction of Pb_4_**1**_4_-G15*b* with 4 equiv. of Zn(OTf)_2_ produces a mixture which no longer contains the grid-like species Pb_4_**1**_4_-G or Zn_4_**1**_4_-G (ESI p. S2†). This suggests that the affinity of Zn^2+^ for the ligand, as well as its preference for octahedral coordination are not sufficient to displace the equilibrium towards Zn_4_**1**_4_-G. We considered that the involvement of Pb^2+^ ions in a weakly dissociating or sparingly soluble compound should displace the equilibrium. Indeed, addition of Br^–^ (as Bu_4_P^+^Br^–^) to the above mixture, or treatment of Pb_4_**1**_4_-G with four equivalents of ZnBr_2_ or ZnCl_2_ produced – along with the formation of PbX_2_ (X = Br, Cl) which precipitates and, doing so, shifts the equilibrium – the expected Zn_4_**1**_4_-G grid (solvent: CD_3_CN; ESI pp. S3–S4†): Pb_4_**1**_4_-G + 4 ZnX_2_ → Zn_4_**1**_4_-G + 4 PbX_2_.

The reverse conversion Zn_4_**1**_4_-G → Pb_4_**1**_4_-G grid was achieved in several steps (Fig. 4b). Treatment of Zn_4_**1**_4_-G (in CD_3_CN) with KOH led to the precipitation of Zn^2+^ (as Zn(OH)_2_ or K_2_[Zn(OH)_4_]), as well as of the free ligand **1**. After removal of CD_3_CN, the free ligand **1** was extracted with CDCl_3_ and used further for the preparation of Pb_4_**1**_4_-G (see ESI, p. S5†).

Thus, in addition to its self-assembly from Zn^2+^ and a ligand, the same Zn^2+^ grid, Zn_4_**1**_4_-G, can be obtained, in reactions (i) and (ii), from a Ag^+^ dinuclear double helicate or from a Pb^2+^ tetranuclear grid (exchange of metal ions and reorganization of the architectures).

(iii) The fusion (conproportionation) reaction of double helicate Ag_2_**1**_2_-DH15*c* with 1 equiv. of Ag_2_**2**_2_-DH (Fig. 5) according to the equationAg_2_**1**_2_-DH + Ag_2_**2**_2_-DH → 2Ag_2_(**1**)(**2**)-DHproduces a mixture that contains each of the three helicates, namely two homoleptic (homostranded) ones and one heteroleptic (heterostranded) one. Ligands **1** and **2** equally participate to homo- and heteroleptic helicates, and so the observed molar percentages are of approximately 25% for Ag_2_**1**_2_-DH, 25% for Ag_2_**2**_2_-DH, and 50% for Ag_2_(**1**)(**2**)-DH. For characterization of the new compound Ag_2_**2**_2_-DH, see ESI pp. S14–S20;† for ^1^H, ^13^C and DOSY of the mixture of three helicates, see ESI pp. S25–S31.†

For the reactions described above it might appear necessary, in practice, to slightly (2–10%) increase the amounts of reagents with respect to those theoretically calculated.

## Experimental

For experimental details, see the ESI.†

## Conclusions

To summarize, three supramolecular reactions were investigated: (i) a Ag_2_**L**_2_-DH double-helicate into Zn_4_**L**_4_-G grid conversion, where the exchange of metal ions changes the nature of the metallo-supramolecular architecture, (ii) a Zn_4_**1**_4_-G grid into Pb_4_**1**_4_-G grid conversion driven by a halide-induced precipitation and where the nature of the metallo-supramolecular architecture is conserved, and (iii) a double exchange of ligands during the fusion of two double helicates.

The grid/grid and double-helicate/grid conversions were made reversible by precipitation of Zn^2+^ with KOH and subsequent reaction of the free ligand with Ag^+^ or Pb^2+^, or, for one DH/G interconversion, in a pH-dependent way.

In perspective, such ligands could be introduced in larger and more complex, suitably decorated, architectures where such supramolecular reactions can act as actuators of various properties (charge, volume, multivalency).

## Supplementary Material

Supplementary information
