# Supplementary material for: Supramolecular reactions of metallo-architectures: Ag2-double-helicate/Zn4-grid, Pb4-grid/Zn4-grid interconversions, and Ag2-double-helicate fusion
Source: Chem Sci. 2016 Feb 29;7(6):3689–93. doi: 10.1039/c5sc04403k (PMC6008726; doi:10.1039/c5sc04403k)

This journal is © The Royal Society of Chemistry 2016

Supporting information for the paper

Supramolecular reactions of metallo-architectures: Ag_2-double-helicate/Zn_4-grid, Pb_4-grid/Zn_4-grid interconversions, and Ag_2-double-helicate fusion

Adrian-Mihail Stadler,[a,b,*] Juan Ramírez,[c] Jean-Marie Lehn,[a] Bruno Vincent[d]

[a] Université de Strasbourg, CNRS, UMR 7006, ISIS, 8 Allée G. Monge, Strasbourg, France
[b] Institute of Nanotechnology (INT), Karlsruhe institute of Technolgoy (KIT), 76344, Eggenstein-Leopoldshafen
[c] Institut Pasteur Paris, 28 Rue du Docteur Roux, 75015 Paris, France
[d] Service de RMN, Faculté de Chimie, 1 Rue B. Pascal, Strasbourg, France
* E-mail : mstadler@unistra.fr

Contents

^{1}H NMR spectra for the reaction: $Pb_4 1_4(OTf)_8$ + 4 $Zn(OTf)_2$ → mixture 2
^{1}H NMR spectra for the reaction: $Pb_4 1_4(OTf)_8$ + 4 $ZnCl_2$ → $Zn_4 1_4(OTf)_8$ + 4 $PbCl_2$ 3
^{1}H NMR spectra for the reaction: $Pb_4 1_4(OTf)_8$ + 4 $ZnBr_2$ → $Zn_4 1_4(OTf)_8$ + 4 $PbBr_2$ 4
^{1}H NMR spectra for the conversion: $Zn_4 1_4(OTf)_8$ → $Pb_4 1_4(OTf)_8$ → $Zn_4 1_4(OTf)_8$ 5
^{1}H NMR and ROESY spectra of complex $Zn_4 1_4(OTf)_8$ in CD_3NO_2 6
^{1}H NMR spectra for the reaction: 2 $Ag_2 1_2(OTf)_2$ + 4 $ZnCl_2$ + 4 AgOTf → $Zn_4 1_4(OTf)_8$ + 8 AgCl 8
^{1}H NMR spectra for the conversion: $Ag_2 1_2(OTf)_2$ → $Zn_4 1_4(OTf)_8$ → $Ag_2 1_2(OTf)_2$ 9
^{1}H NMR spectra for the conversion: $Ag_2 1_2(OTf)_2$ → $Zn_4 1_4(OTf)_8$ → $Ag_2 1_2(OTf)_2$ → $Zn_4 1_4(OTf)_8$ → $Ag_2 1_2(OTf)_2$ 10
^{1}H NMR spectra for the reaction: 2 $Ag_2 2_2(OTf)_2$ + 4 $Zn(OTf)_2$ → $Zn_4 2_4(OTf)_8$ + 4 AgOTf 11
^{1}H NMR spectra for the reactions: 2 **2** + 2 AgOTf → $Ag_2 2_2(OTf)_2$
2 $Ag_2 2_2(OTf)_2$ + 4 $Zn(OTf)_2$ → $Zn_4 2_4(OTf)_8$ + 4 AgOTf 12
^{1}H NMR spectra for the conversion: $Zn_4 2_4(OTf)_8$ → $Ag_2 2_2(OTf)_2$ 13
Complex $Ag_2 2_2(OTf)_2$ 14
Complex $Ag_2 1_2(OTf)_2$ 21
The reaction mixture $Ag_2 1_2(OTf)_2$ + $Ag_2 2_2(OTf)_2$ → 2 $Ag_2(1)(2)(OTf)_2$ 25

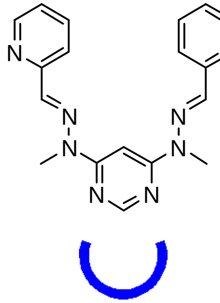

1

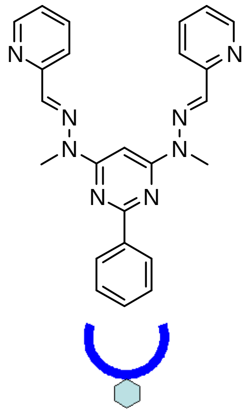

2

For the complexes, the notations of type $M_nL_n(OTf)_m$, $[M_nL_n]^{m+}$ and M_nL_n are to be considered equivalent.
"G" and "DH" were omitted from the notations of the complexes.

$CDCl_3$ and CD_3NO_2 were filtered through basic alumina.

^{1}H NMR spectra for the reaction: $Pb_41_4(OTf)_8 + 4\ Zn(OTf)_2 \rightarrow$ mixture

(400 MHz, CD_3CN)

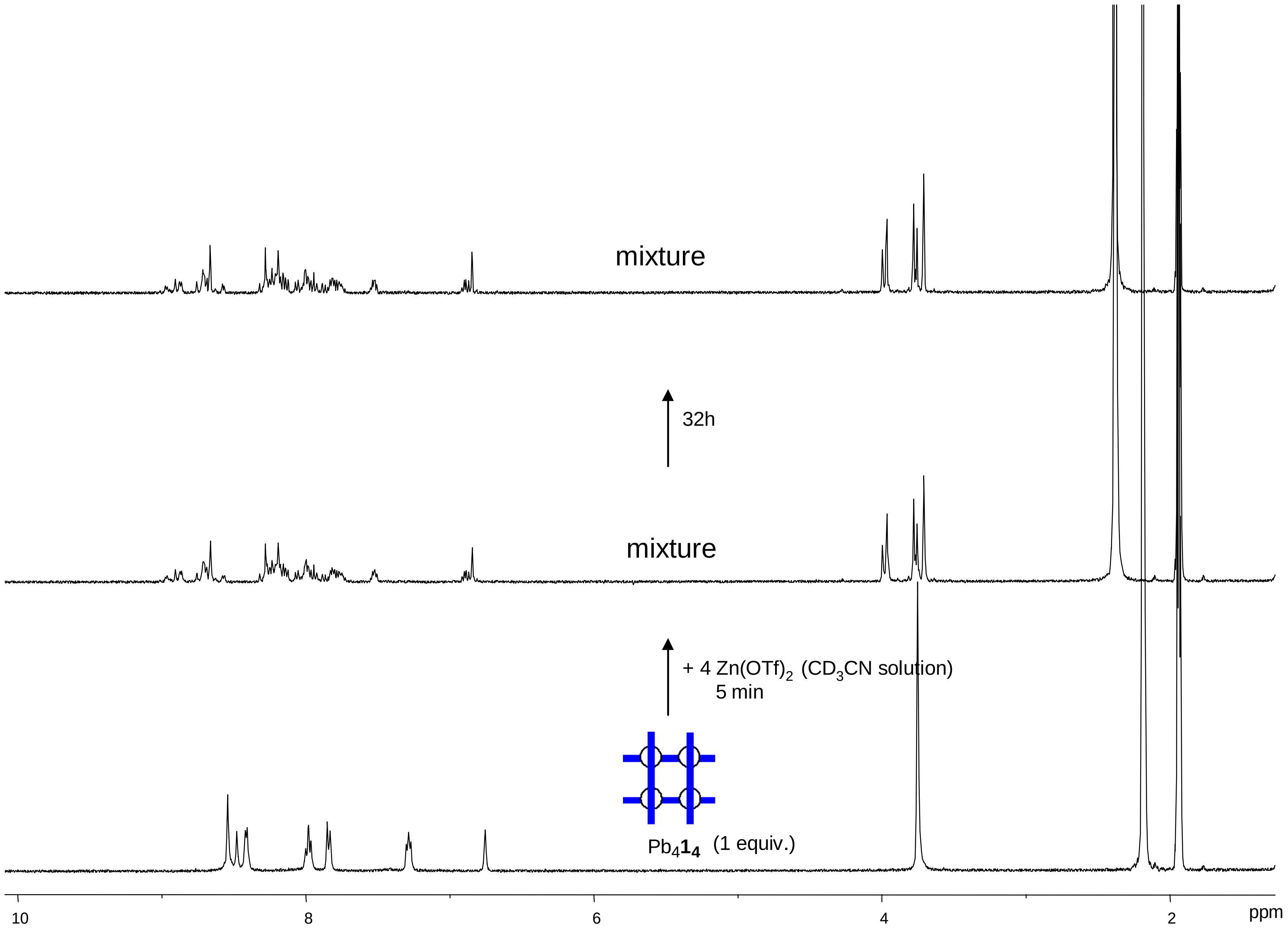

^{1}H NMR spectra for the reaction: $Pb_41_4(OTf)_8 + 4\ ZnCl_2 \longrightarrow Zn_41_4(OTf)_8 + 4\ PbCl_2$

(400 MHz, CD_3CN)

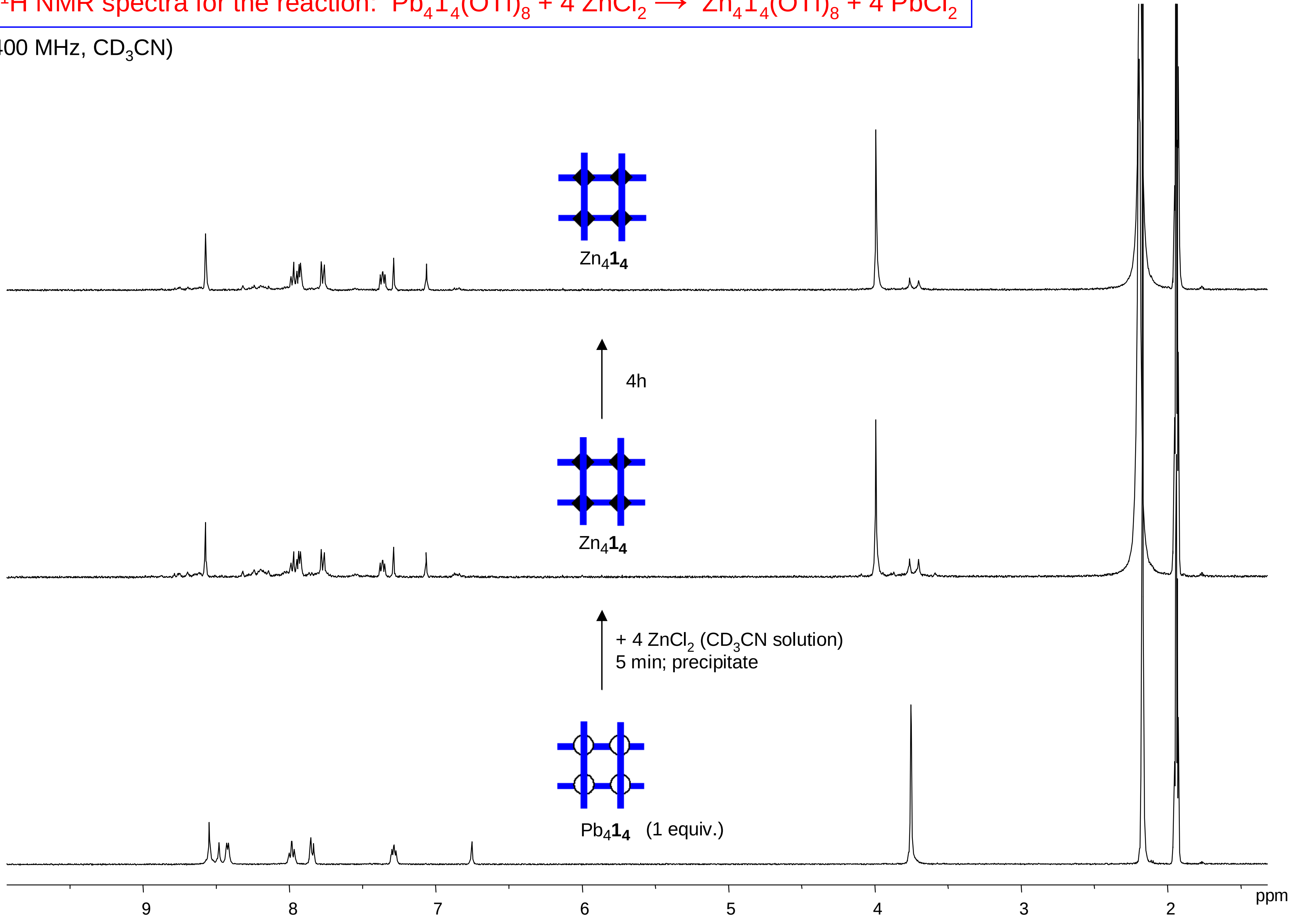

^{1}H NMR spectra for the reaction: $Pb_4 1_4(OTf)_8 + 4\ ZnBr_2 \longrightarrow Zn_4 1_4(OTf)_8 + 4\ PbBr_2$

(400 MHz, CD_3CN)

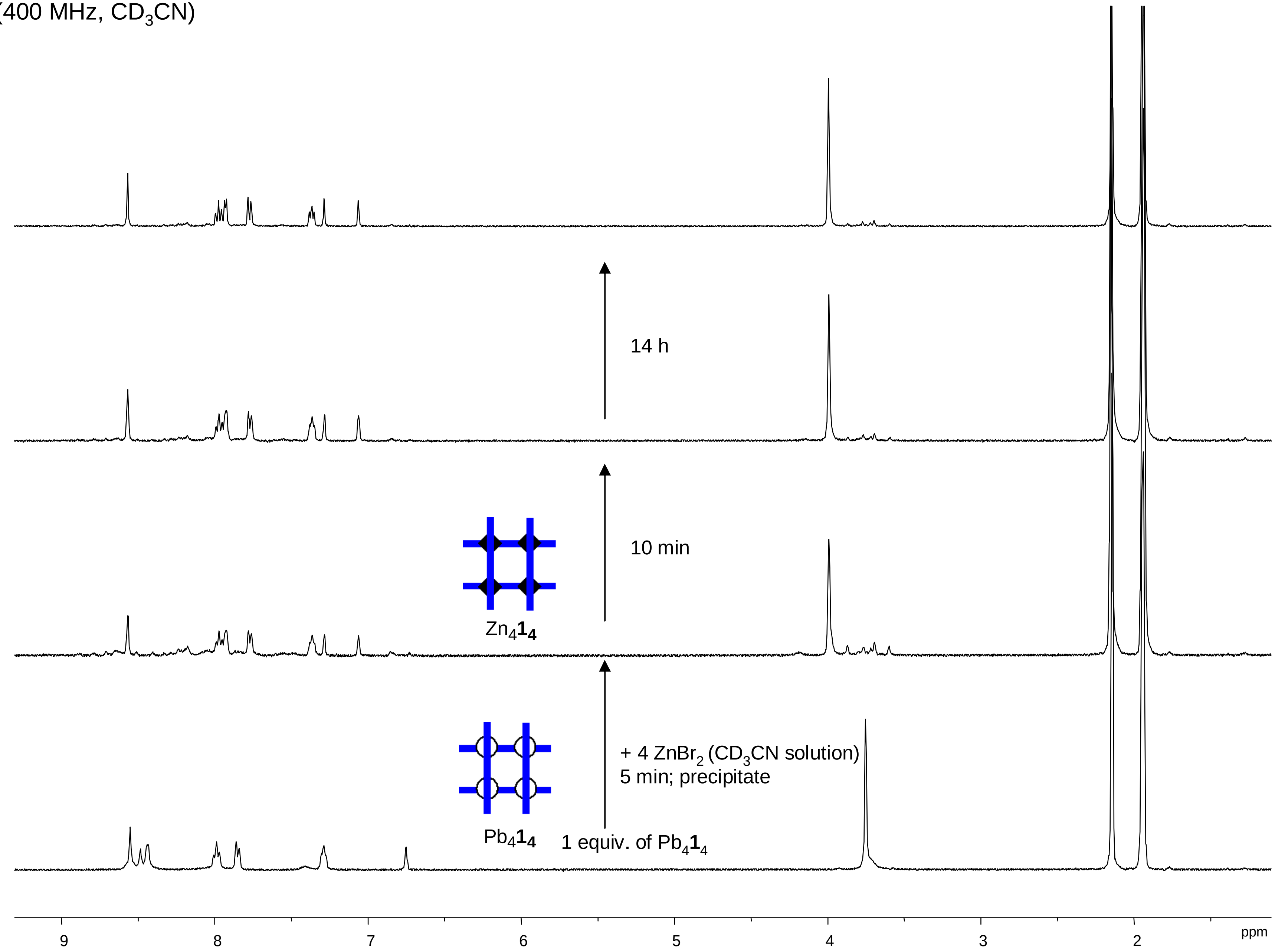

^{1}H NMR spectra for the conversion: $Zn_4\mathbf{1}_4(OTf)_8 \rightarrow Pb_4\mathbf{1}_4(OTf)_8 \rightarrow Zn_4\mathbf{1}_4(OTf)_8$

(400 MHz, starting solvent: CD_3CN)

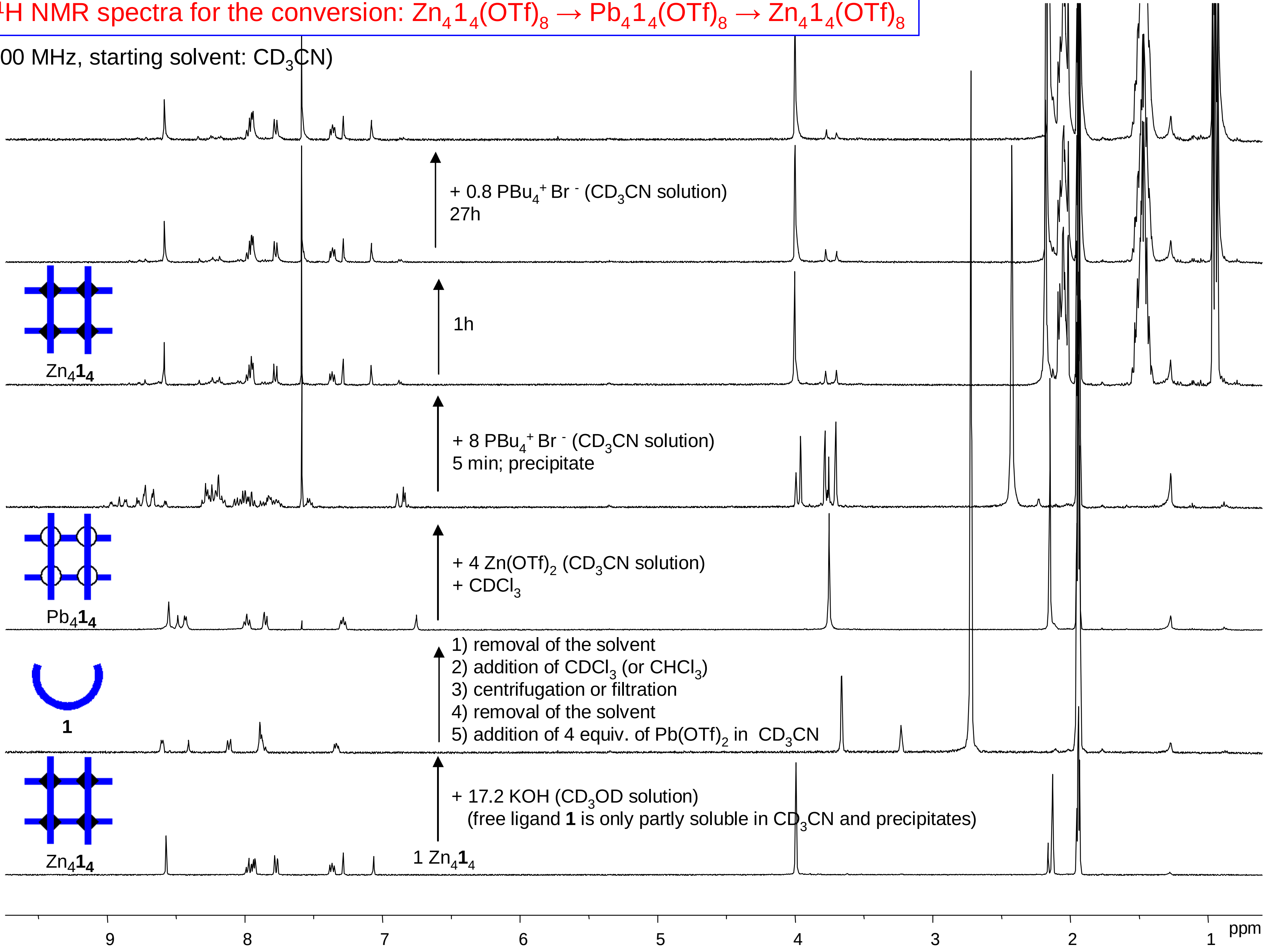

^{1}H NMR spectrum of complex $Zn_4\mathbf{1}_4(OTf)_8$

(400 MHz, CD_3NO_2)

Reference: A.-M. Stadler, N. Kyritsakas, R. Graff and J.-M. Lehn, *Chem.-Eur. J*, 2006, **12**, 4503-4522.

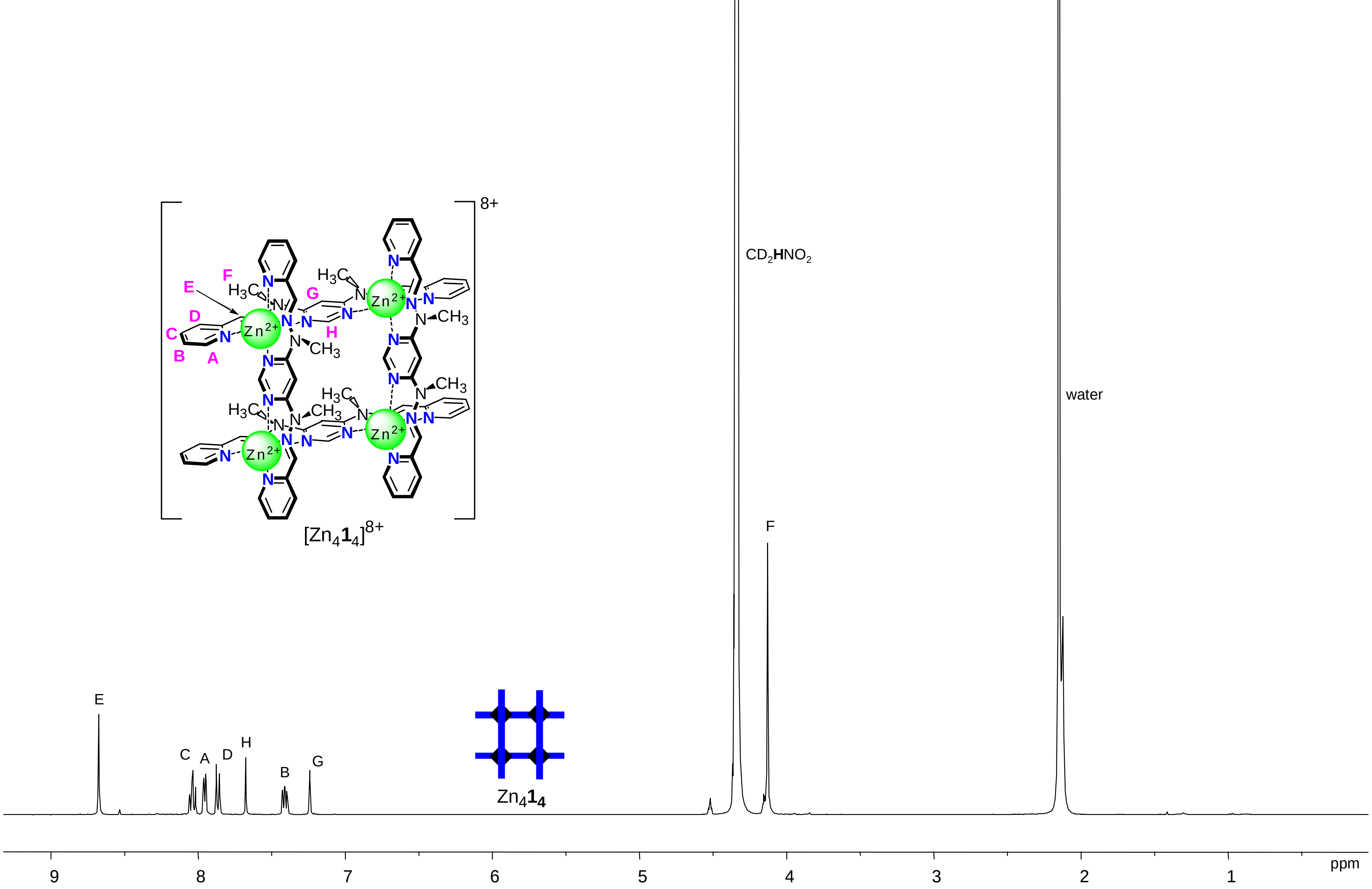

^1H-^1H ROESY spectrum (400 MHz, CD_3NO_2) of compound $Zn_41_4(OTf)_8$

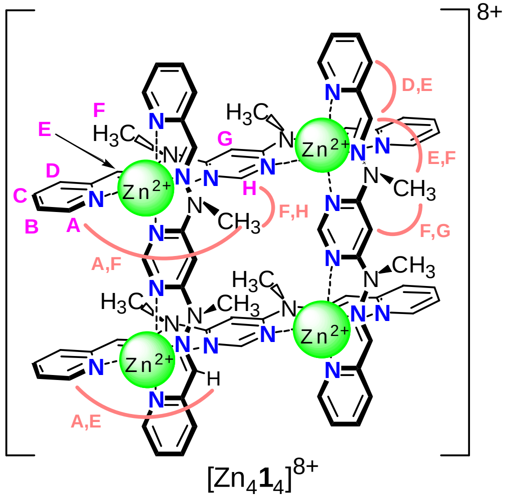

$[Zn_4\mathbf{1}_4]^{8+}$

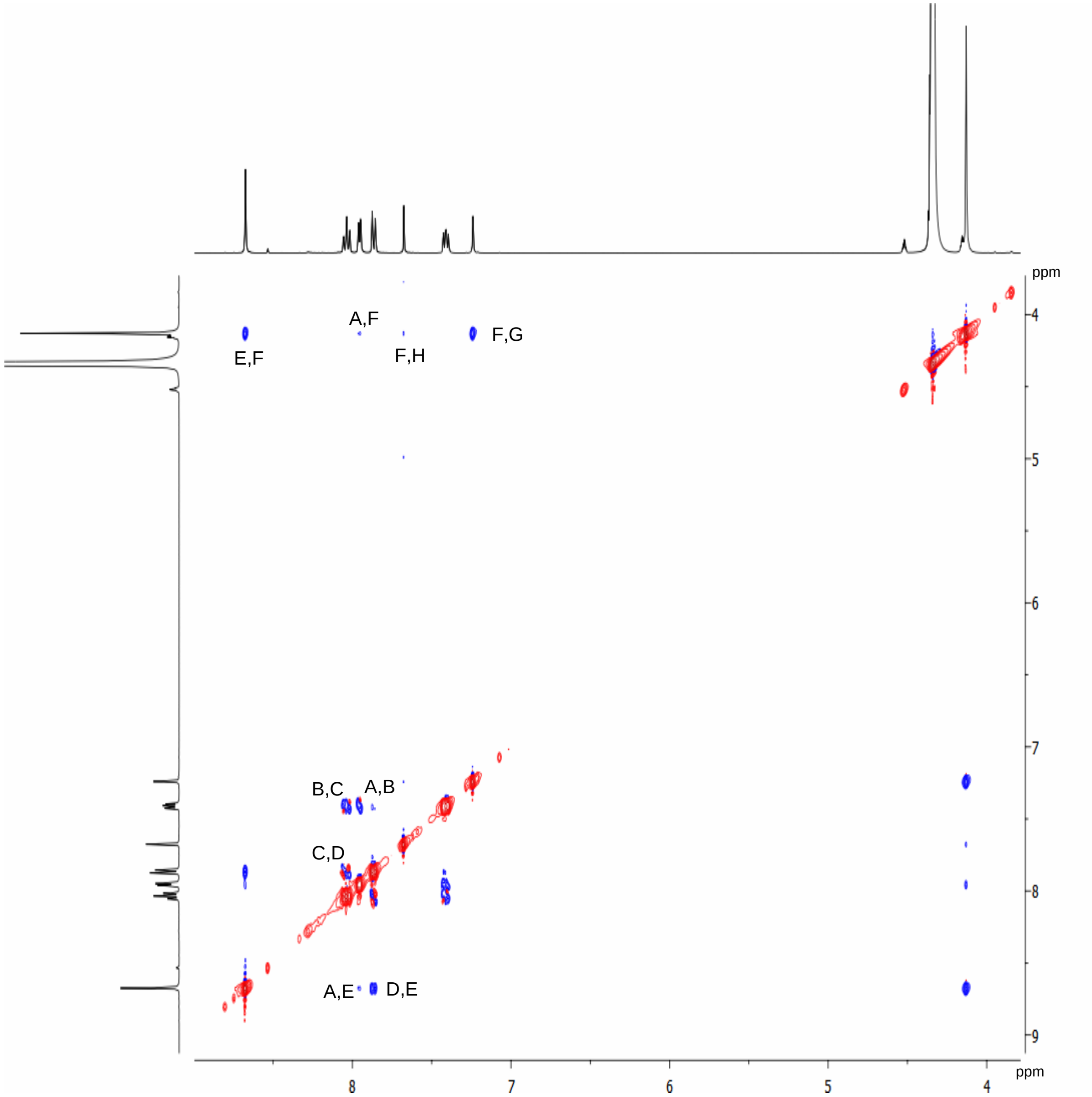

^{1}H NMR spectra for the reaction: 2 $Ag_21_2(OTf)_2$ + 4 $ZnCl_2$ + 4 AgOTf ⟶ $Zn_41_4(OTf)_8$ + 8 AgCl

(400 MHz, starting solvent: CD_3NO_2/CD_3CN 18/1)

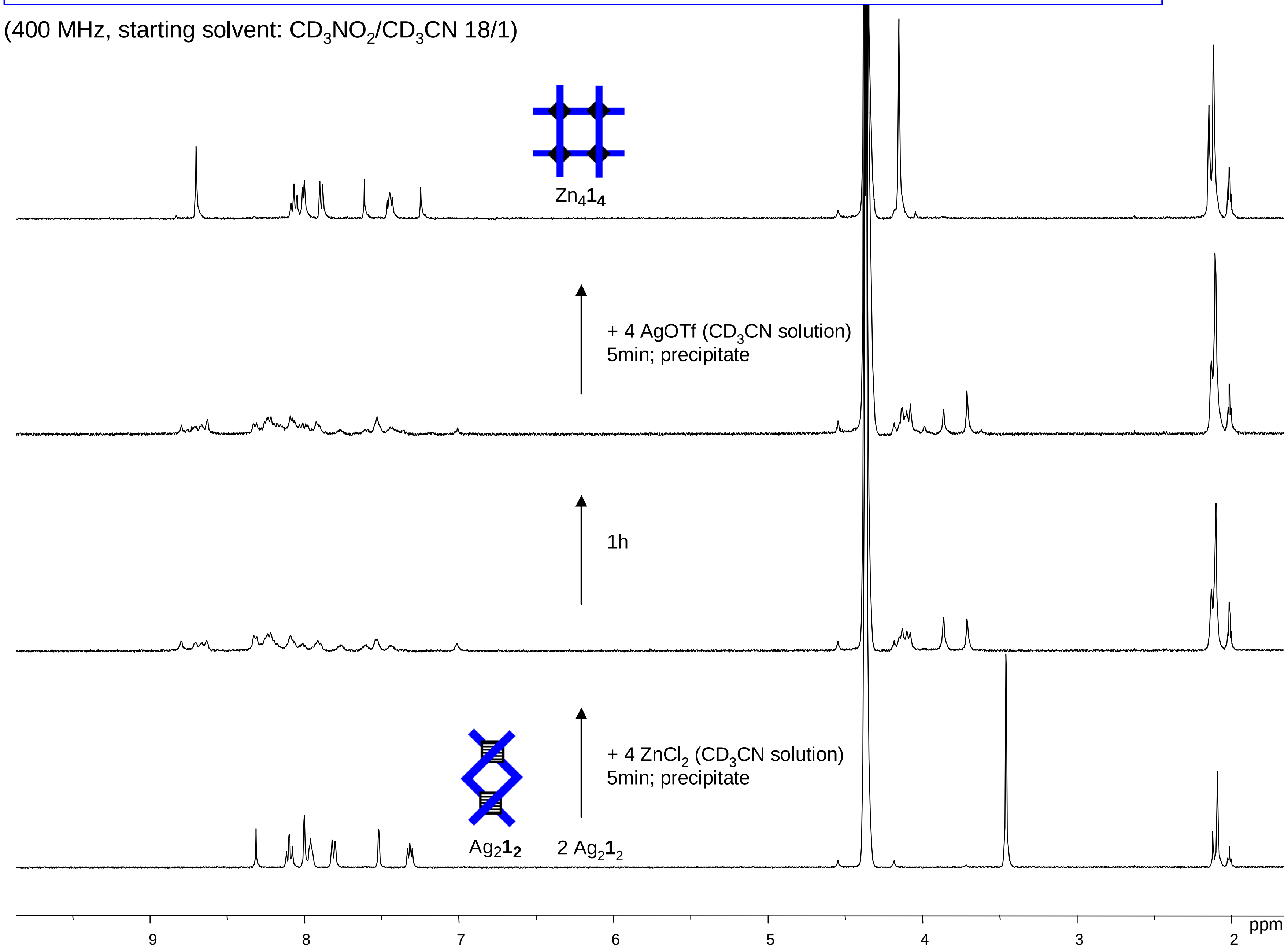

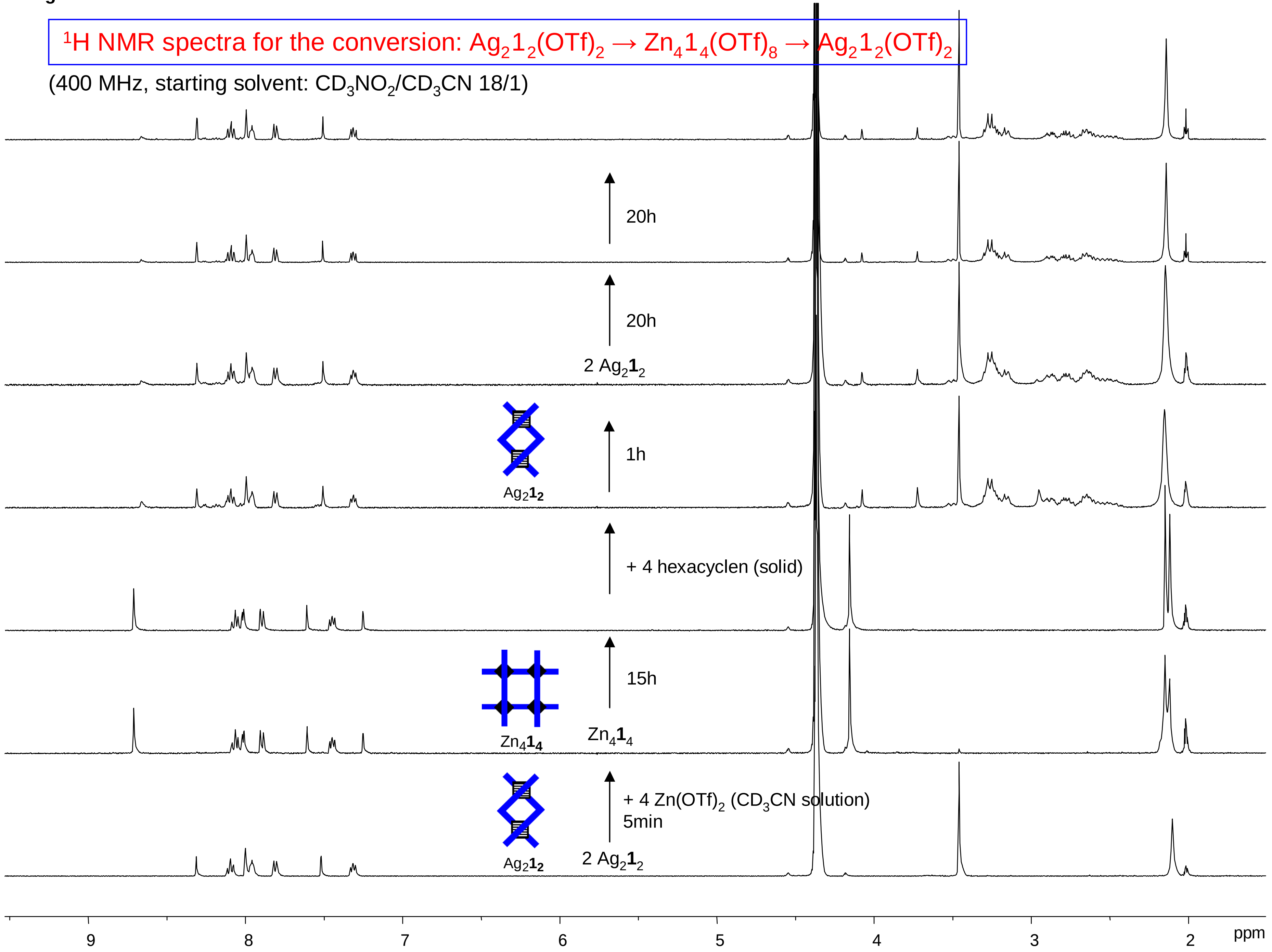

^1H NMR spectra for the conversion: $Ag_2 1_2(OTf)_2 \rightarrow Zn_4 1_4(OTf)_8 \rightarrow Ag_2 1_2(OTf)_2$
(400 MHz, starting solvent: CD_3NO_2/CD_3CN 18/1)
20h
20h
2 $Ag_2\mathbf{1}_2$
1h
$Ag_2\mathbf{1}_2$
+ 4 hexacyclen (solid)
15h
$Zn_4\mathbf{1}_4$
$Zn_4\mathbf{1}_4$
+ 4 $Zn(OTf)_2$ (CD_3CN solution)
5min
$Ag_2\mathbf{1}_2$
2 $Ag_2\mathbf{1}_2$
9
8
7
6
5
4
3
2
ppm

^{1}H NMR spectra for the conversion: $Ag_21_2(OTf)_2$ → $Zn_41_4(OTf)_8$ → $Ag_21_2(OTf)_2$ → $Zn_41_4(OTf)_8$ → $Ag_21_2(OTf)_2$

(400 MHz, starting solvent: CD_3NO_2/CD_3CN 55/1)

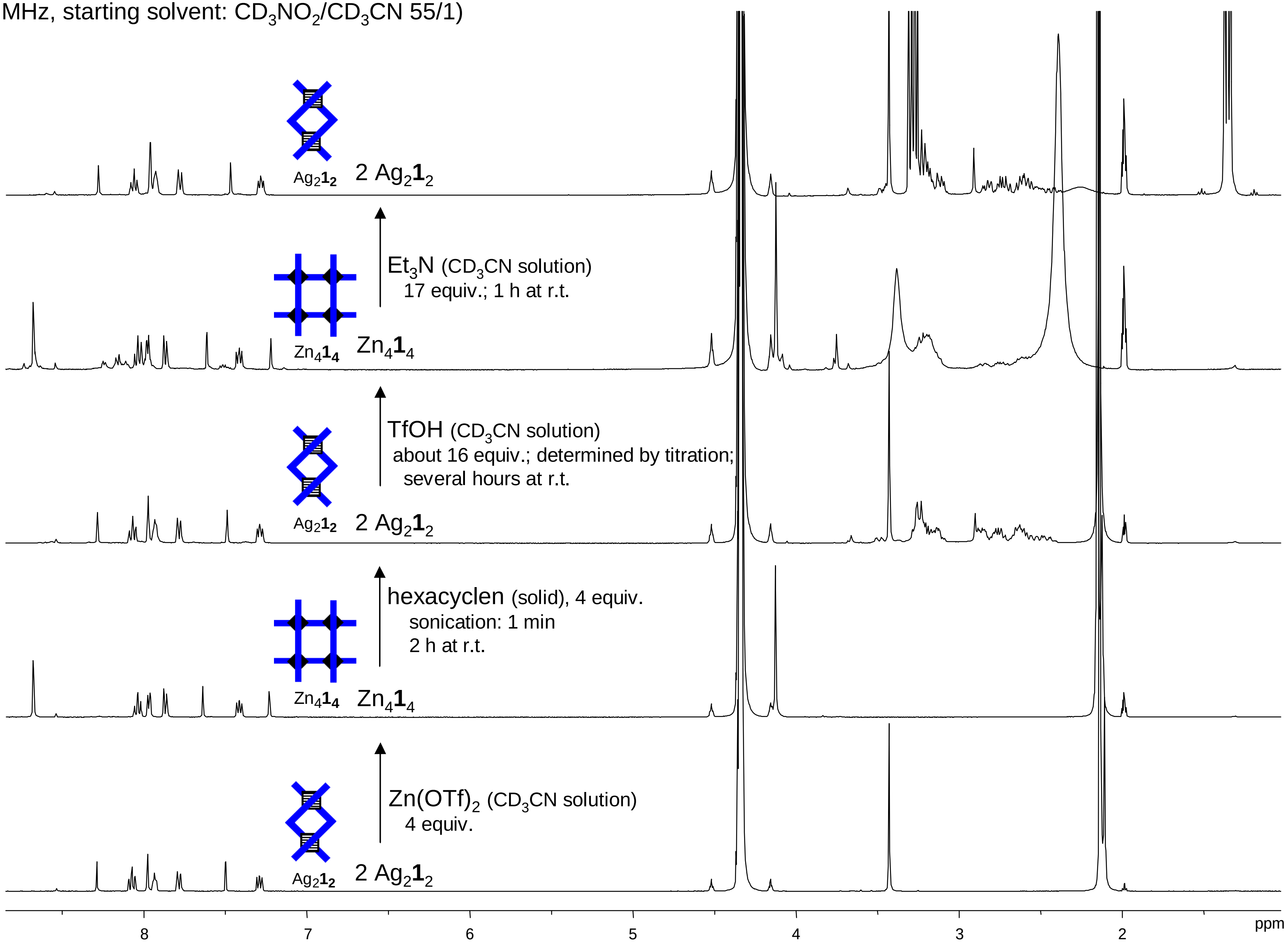

^{1}H NMR spectra for the reaction: 2 Ag_2**2**$_2(OTf)_2$ + 4 $Zn(OTf)_2$ → Zn_4**2**$_4(OTf)_8$ + 4 AgOTf

(400 MHz, starting solvent: CD_3NO_2/CD_3CN 18/1)

Reference for Zn_4**2**$_4(OTf)_8$: M. Barboiu, M. Ruben, G. Blasen, N. Kyritsakas, E. Chacko, M. Dutta, O. Radekovich, K. Lenton, D. J. R. Brook and J.-M. Lehn, *Eur. J. Inorg. Chem.*, 2006, 784–789

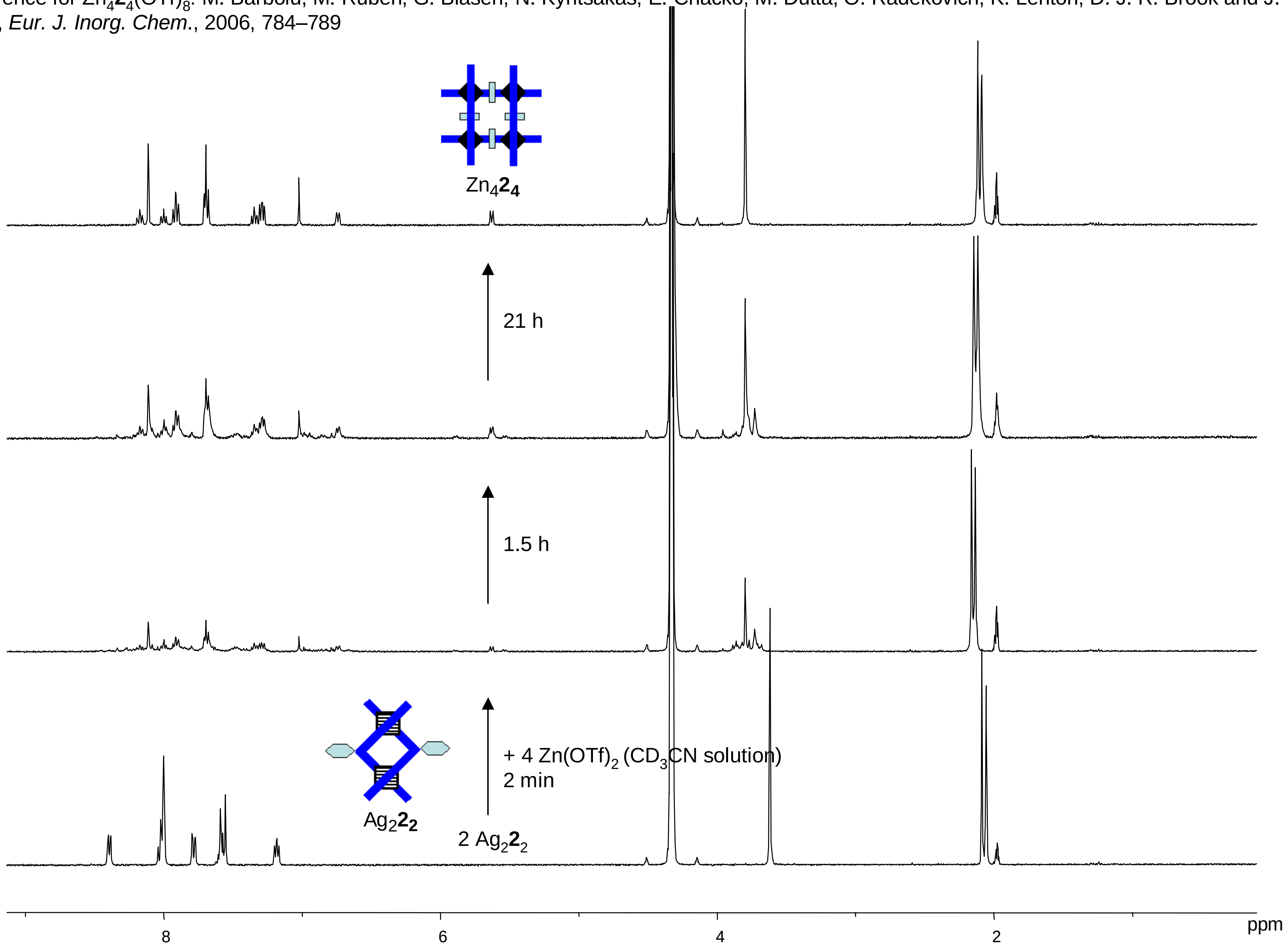

^{1}H NMR spectra for the reactions: 2 **2** + 2 AgOTf → $Ag_2\mathbf{2}_2(OTf)_2$

2 $Ag_2\mathbf{2}_2(OTf)_2$ + 4 $Zn(OTf)_2$ → $Zn_4\mathbf{2}_4(OTf)_8$ + 4 AgOTf

(400 MHz, starting solvent: $CDCl_3/CD_3NO_2$ 1/1)

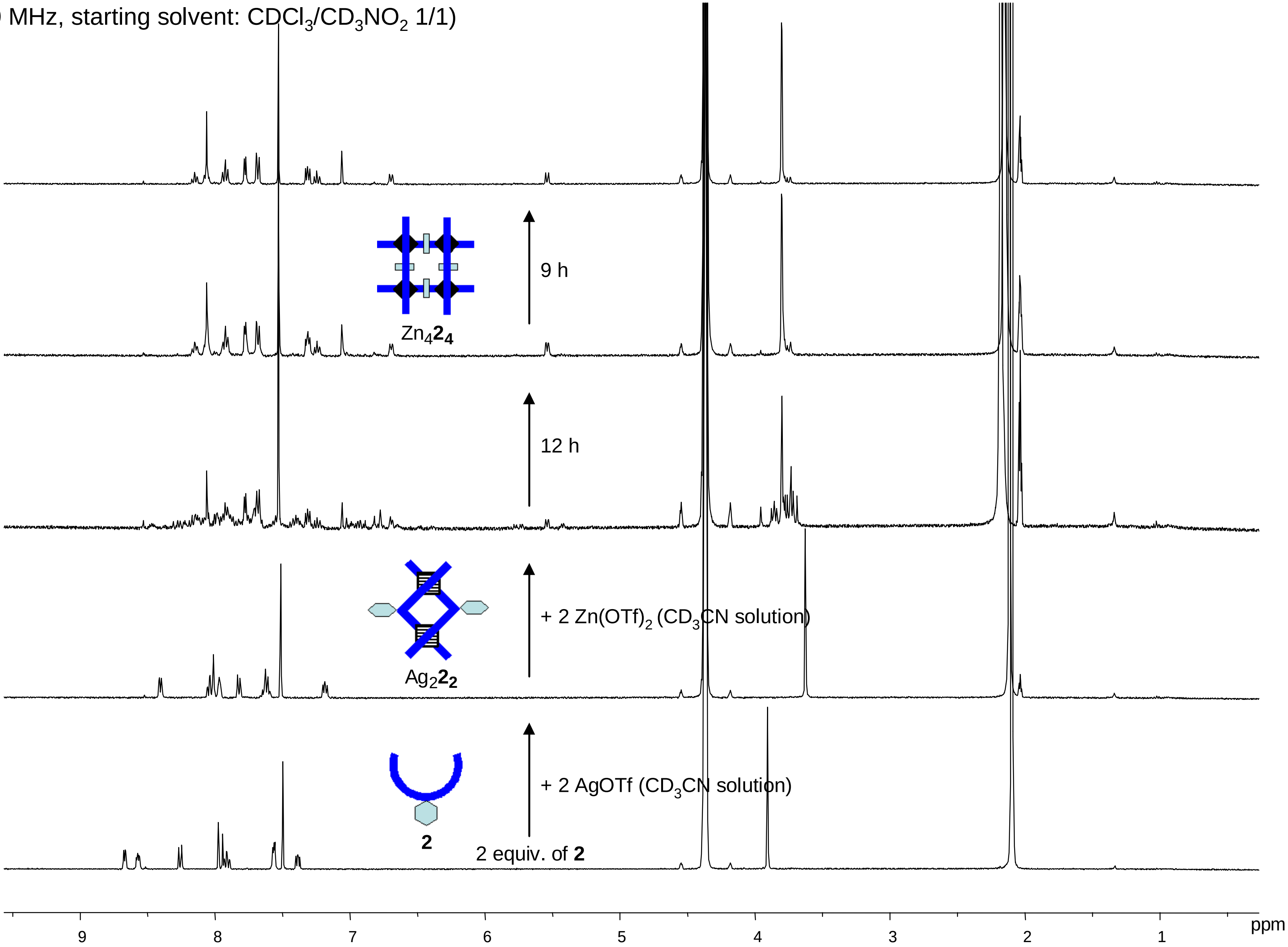

^{1}H NMR spectra for the conversion: $Zn_4 2_4(OTf)_8 \rightarrow Ag_2 2_2(OTf)_2$

(400 MHz, starting solvent: $CDCl_3/CD_3NO_2$ 1/1)

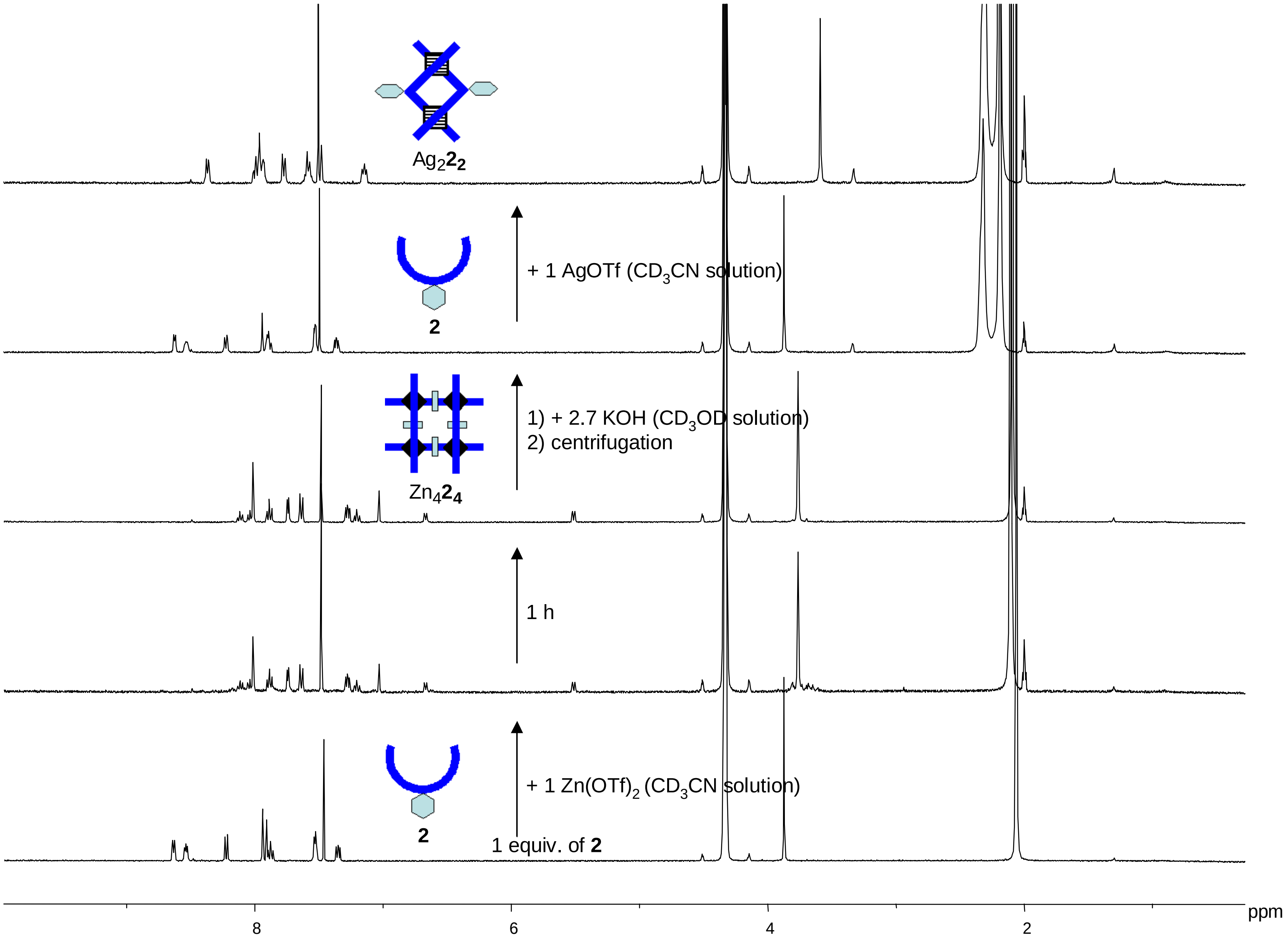

Complex $Ag_2\mathbf{2}_2(OTf)_2$

To a suspension of ligand **2** (1.06 mg, 1 equiv) in CD_3NO_2 (0.55 mL) was added $AgCF_3SO_3$ (1 equiv) in CD_3CN (34 µL) and the mixture was sonicated for 1 min at r.t. Pale yellow solution. ^{1}H NMR (400 MHz, $CD_3NO_2/CD_3CN \approx 16/1$ (v/v), reference C***H***D_2NO_2 peak, δ_{ref} = 4.34 ppm): 8.40 (dd, *J* = 7.8, 1.8 Hz, 4H), 8.06 – 7.97 (m, 12H), 7.81 – 7.75 (m, 4H), 7.64 – 7.54 (m, 8H), 7.22 – 7.15 (m, 4H), 3.63 (s, 12H) ppm; ^{13}C NMR (101 MHz, $CD_3NO_2/CD_3CN \approx 16/1$ (v/v), reference ***C***D_3NO_2 peak, δ_{ref} = 62.9 ppm): 163.9, 163.4, 152.8, 152.0, 141.1, 138.9, 138.3 (d, *J* = 3.3 Hz), 132.5, 129.9, 129.6, 127.5 (d, *J* = 3.9 Hz), 126.9 (d, *J* = 3.6 Hz), 90.9, 32.3 ppm. ESI-MS (m/z): $[C_{49}H_{44}F_3N_{16}O_3SAg_2]^+ = [Ag_2\mathbf{2}_2OTf]^+$, calcd. 1209.16, found 1209.15.

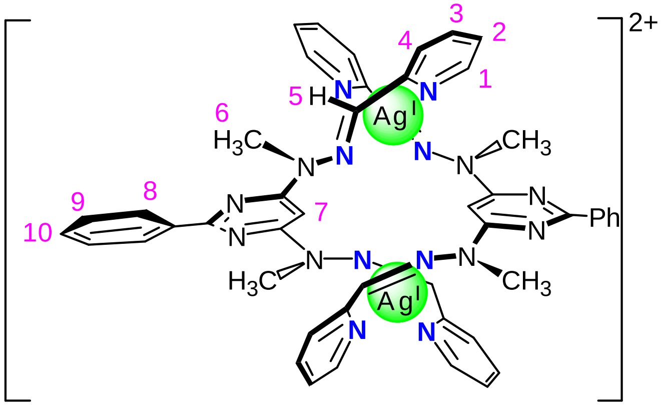

$[Ag_2\mathbf{2}_2]^{2+}$

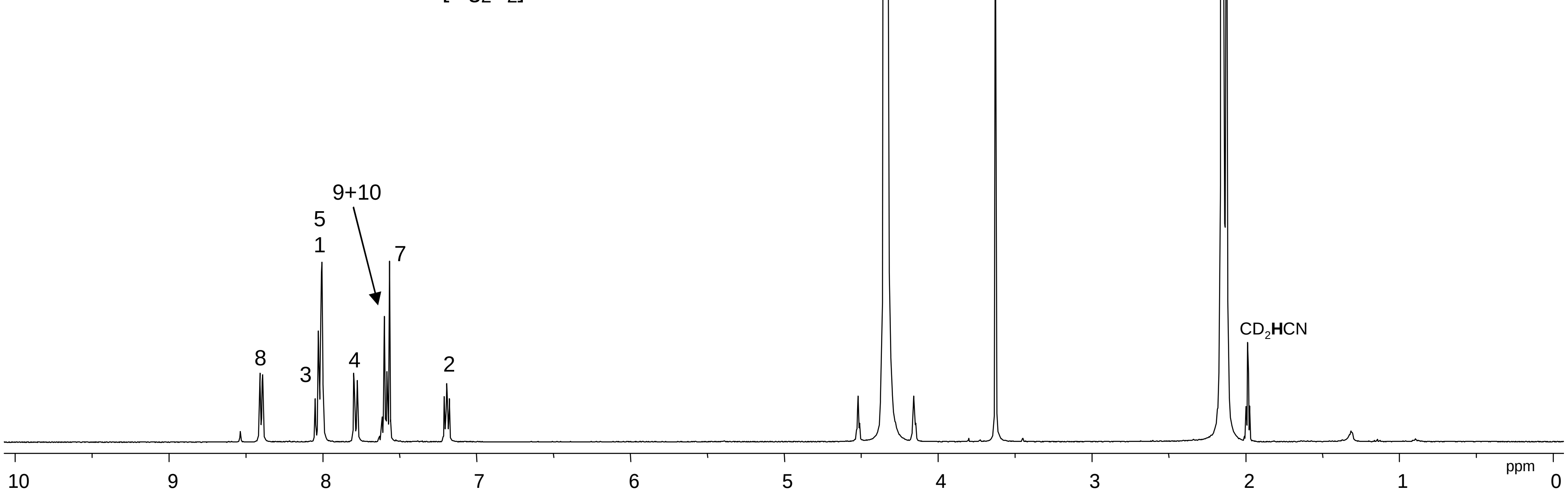

^{1}H-^{1}H COSY spectrum (400 MHz, $CD_3NO_2/CD_3CN \approx 16/1$ (v/v)) of compound $Ag_2\mathbf{2}_2(OTf)_2$

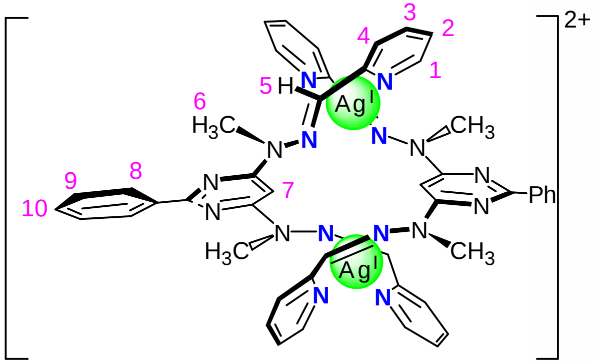

$[Ag_2\mathbf{2}_2]^{2+}$

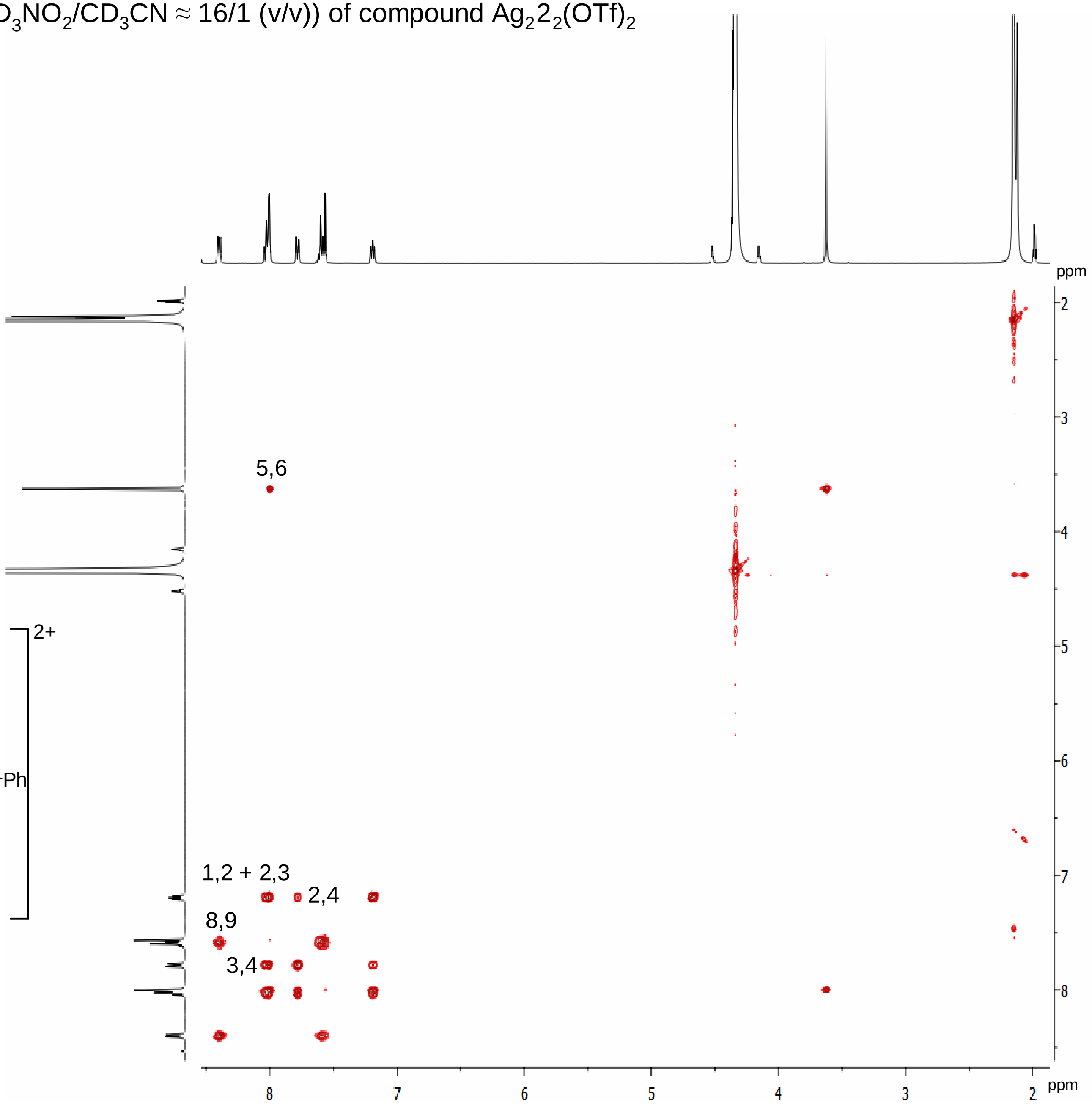

^{1}H-^{1}H NOESY spectrum (400 MHz, CD_3NO_2) of compound $Ag_22_2(OTf)_2$

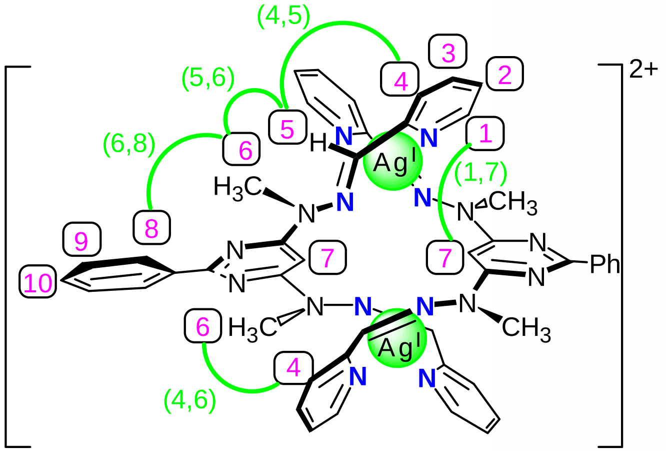

$[Ag_2\mathbf{2}_2]^{2+}$

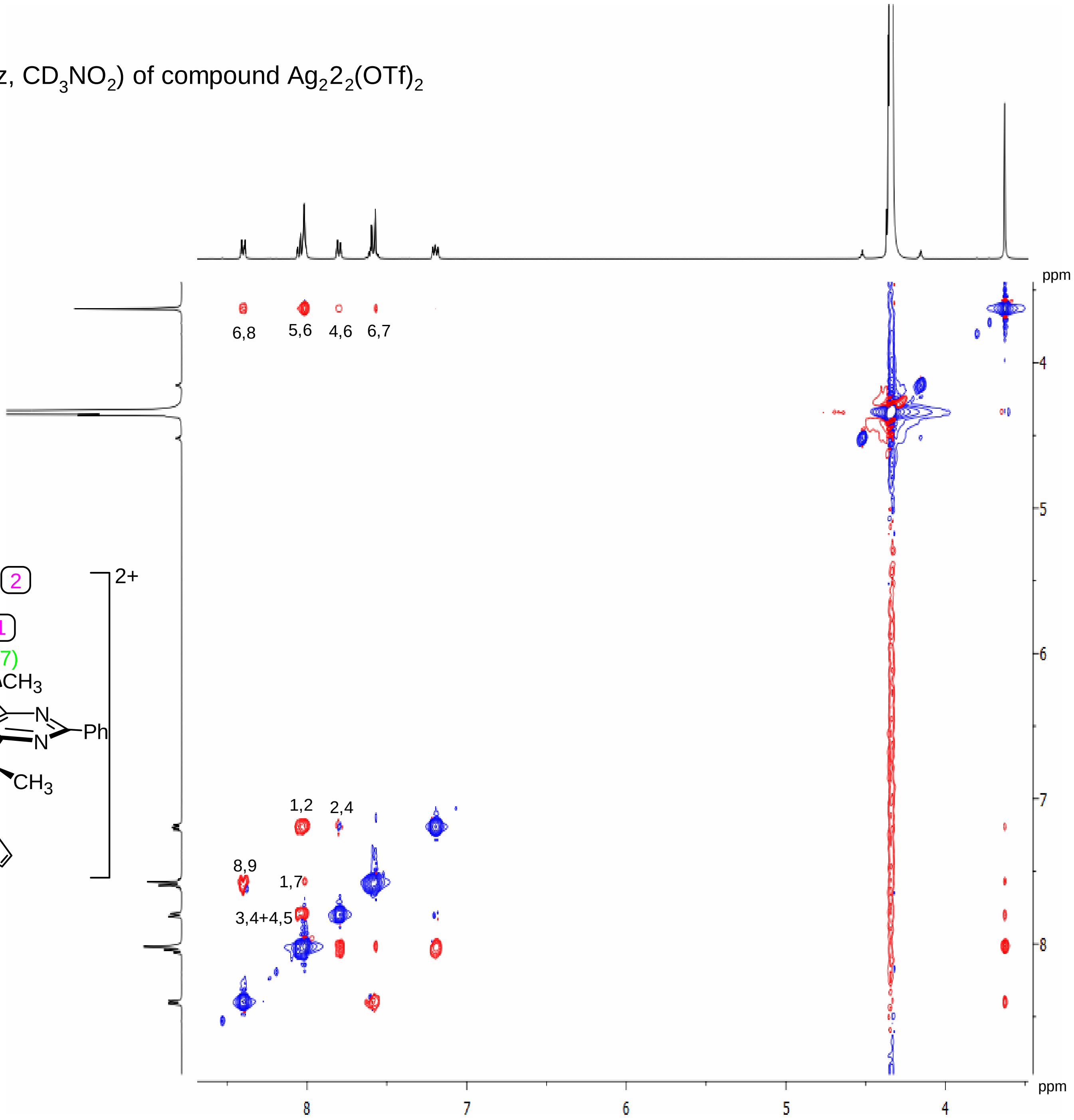

^{1}H-^{13}C HSQC spectrum (400 MHz, $CD_3NO_2/CD_3CN \approx 16/1$ (v/v)) of compound $Ag_22_2(OTf)_2$

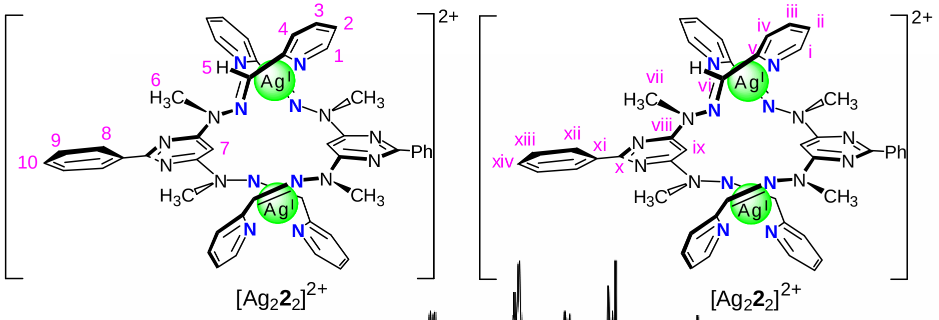

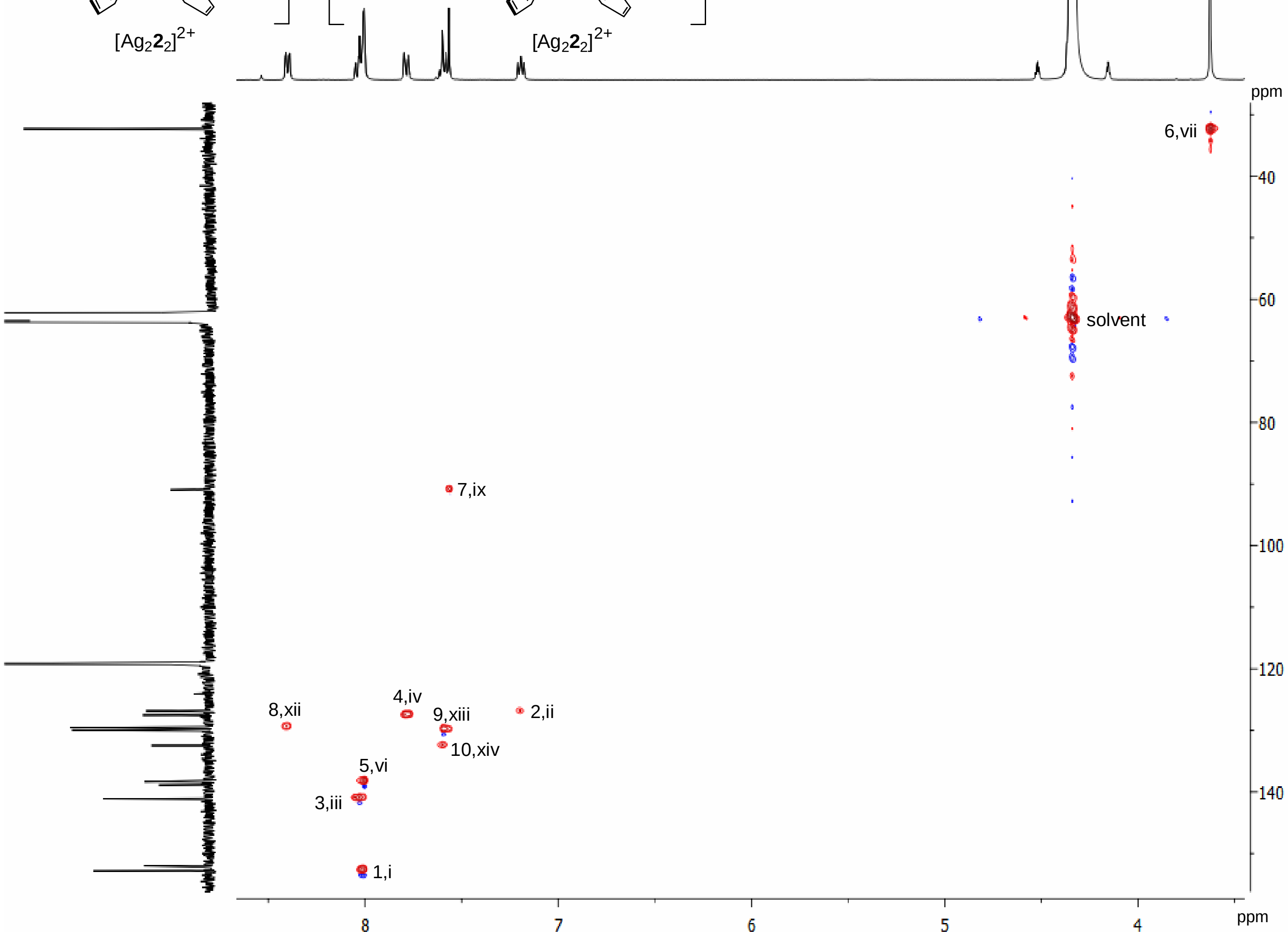

^{1}H-^{13}C HMBC spectrum (400 MHz, $CD_3NO_2/CD_3CN \approx 16/1$ (v/v)) of compound $Ag_22_2(OTf)_2$

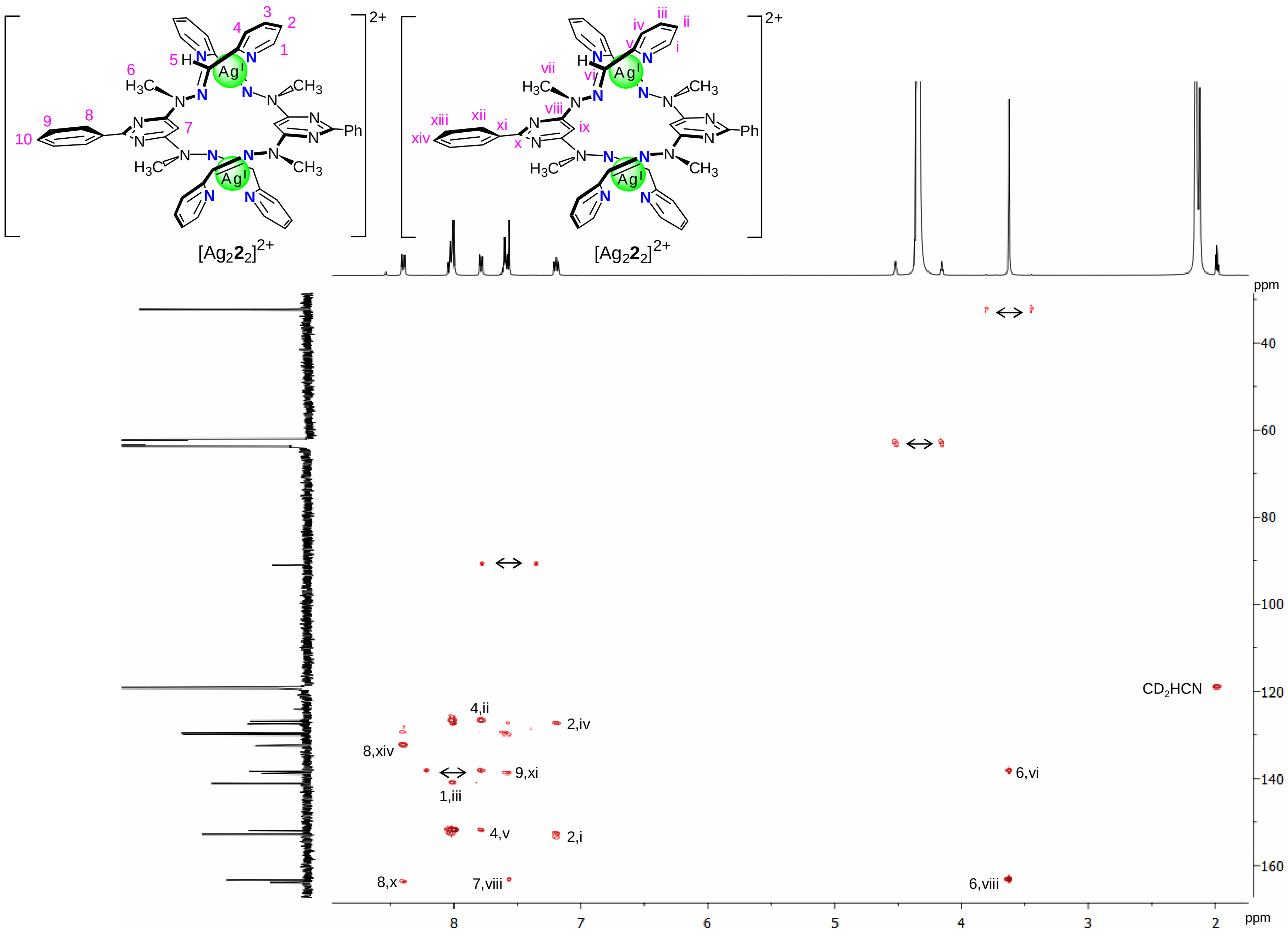

^{13}C NMR spectrum (101 MHz, $CD_3NO_2/CD_3CN \approx 16/1$ (v/v)) of compound $Ag_2\mathbf{2}_2(OTf)_2$

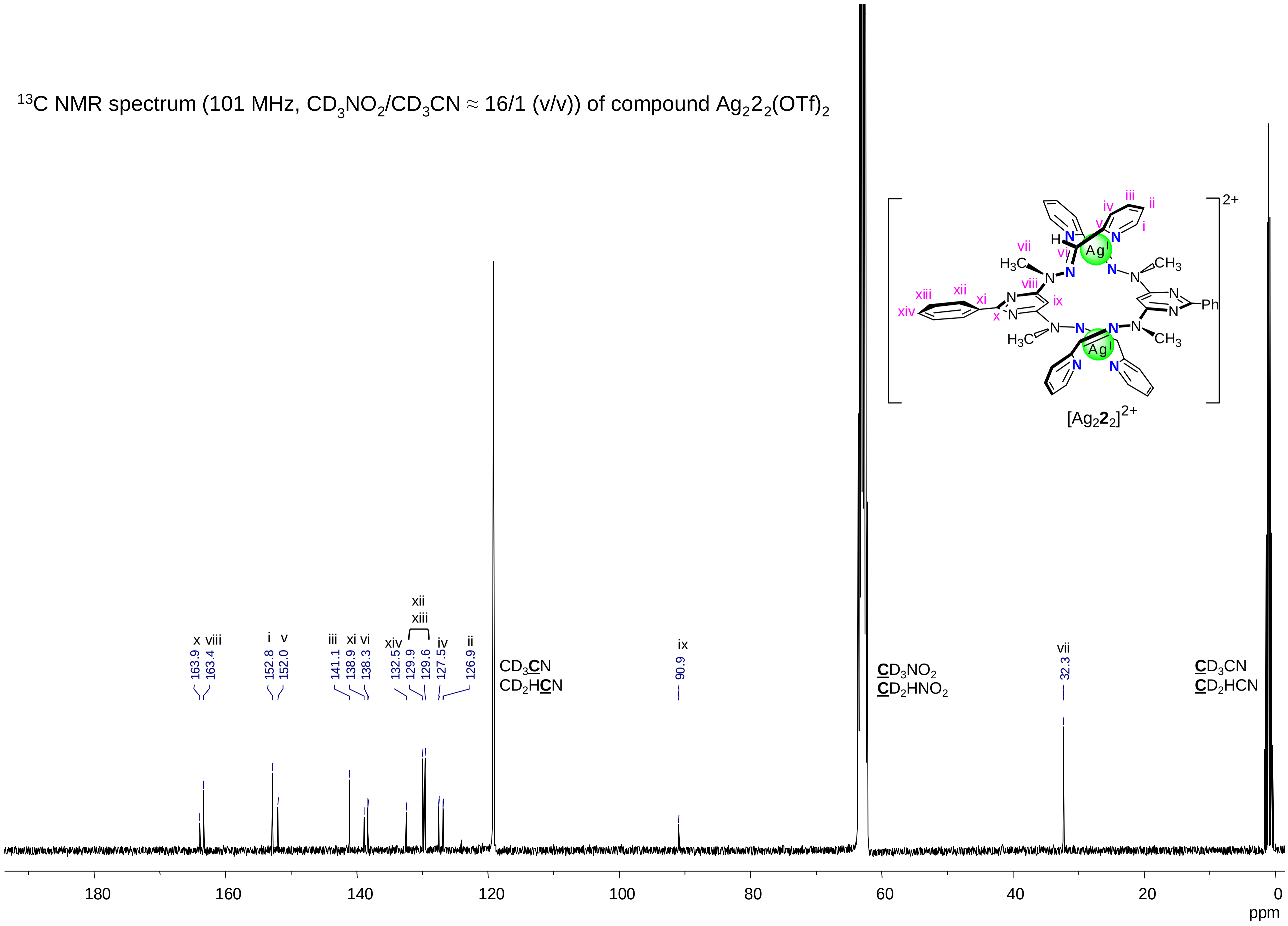

Comparison between the calculated (bottom) and the observed (top) MS peak of [Ag_2**2**$_2$OTf]$^+$

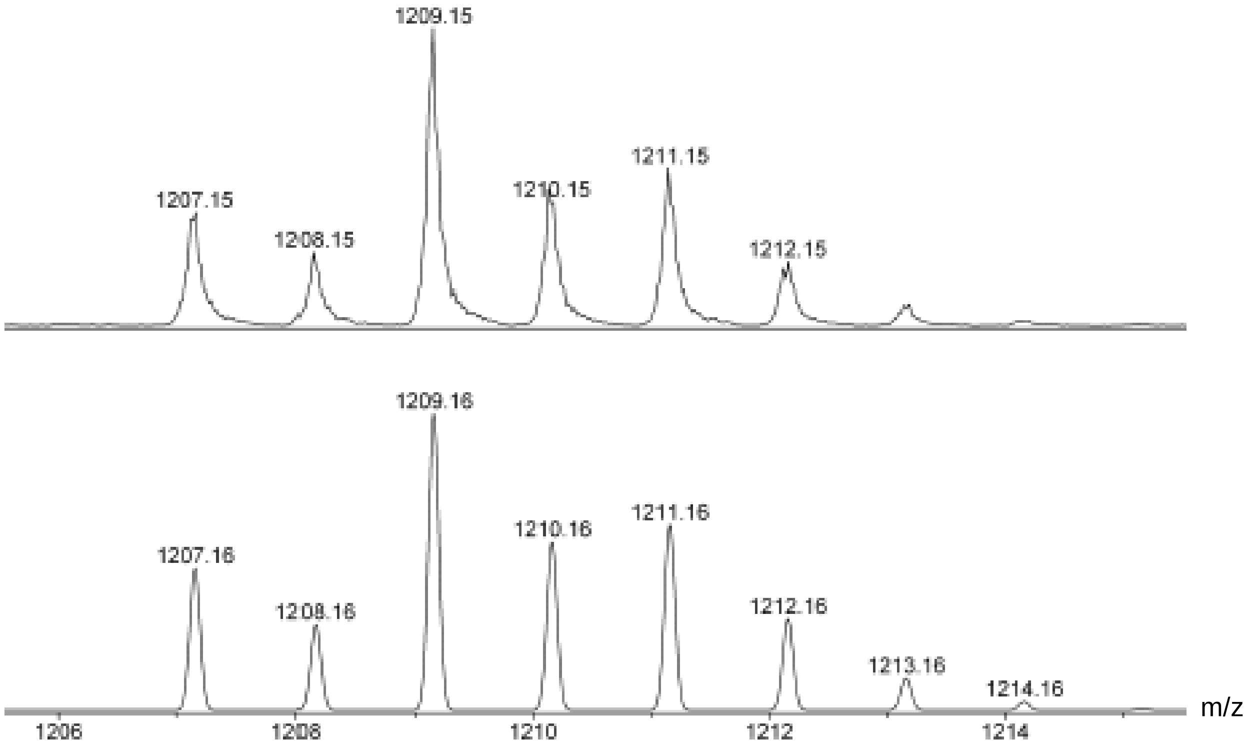

Complex $Ag_21_2(OTf)_2$

^{1}H NMR spectrum (400 MHz, $CD_3NO_2/CD_3CN \approx 28/1$ (v/v)) of compound $Ag_21_2(OTf)_2$

Reference: A.-M. Stadler, N. Kyritsakas, G.Vaughan and J.-M. Lehn, *Chem. Eur. J.* 2007, **13**, 59-68.

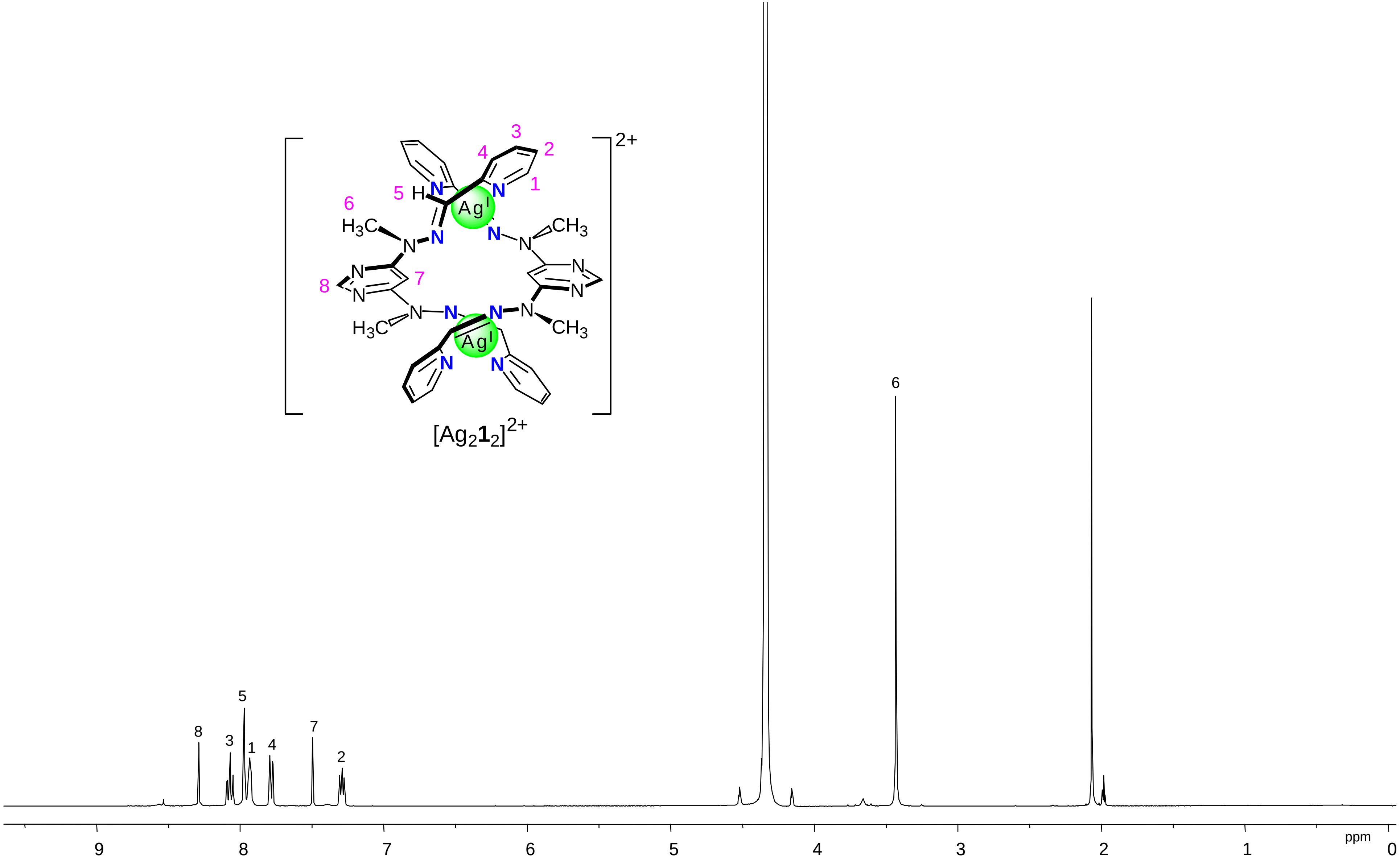

^{1}H-^{13}C HSQC spectrum (400 MHz, $CD_3NO_2/CD_3CN \approx 28/1$ (v/v)) of compound $Ag_2\mathbf{1}_2(OTf)_2$

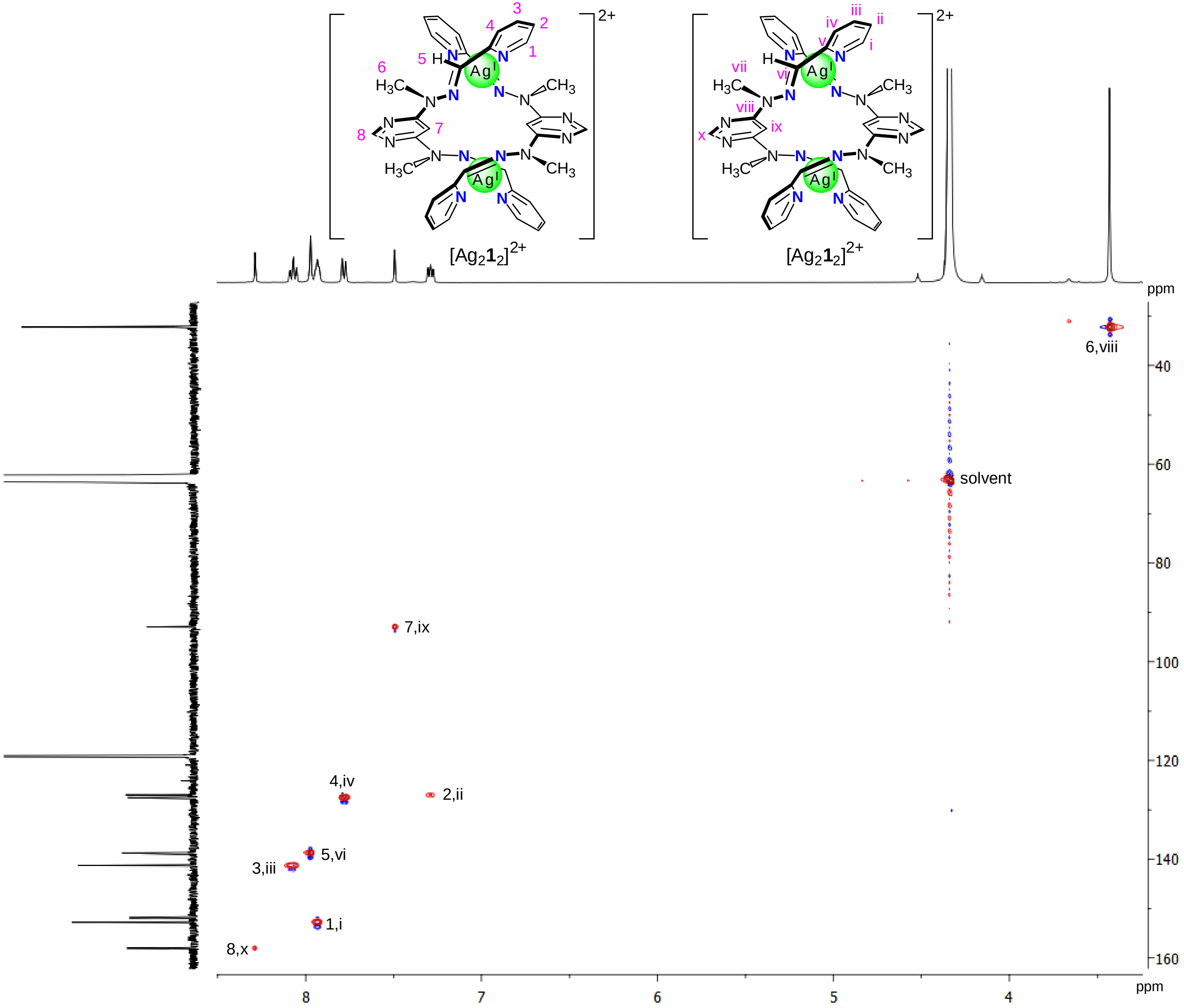

^{1}H-^{13}C HMBC spectrum (400 MHz, $CD_3NO_2/CD_3CN \approx 28/1$ (v/v)) of compound $Ag_21_2(OTf)_2$

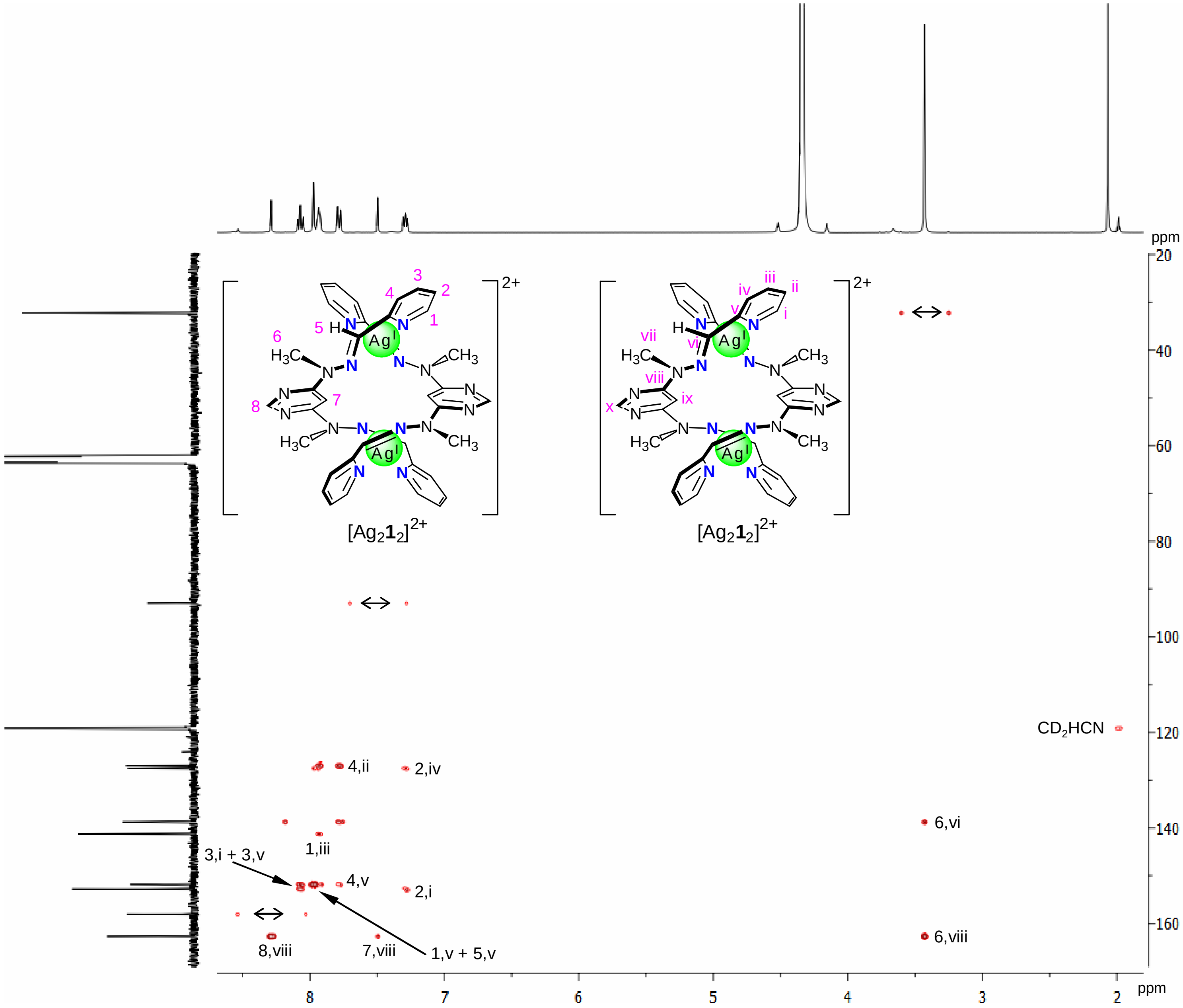

^{13}C NMR spectrum of compound $Ag_2\mathbf{1}_2(OTf)_2$ (101 MHz, $CD_3NO_2/CD_3CN \approx 28/1$ (v/v), reference $\mathbf{C}D_3NO_2$ peak, δ_{ref} = 62.9 ppm): 162.7, 158.0, 152.8, 151.9, 141.3, 138.8 (d, J = 3.4 Hz), 127.5 (d, J = 3.9 Hz), 127.0 (d, J = 3.6 Hz), 93.0, 32.2 ppm.

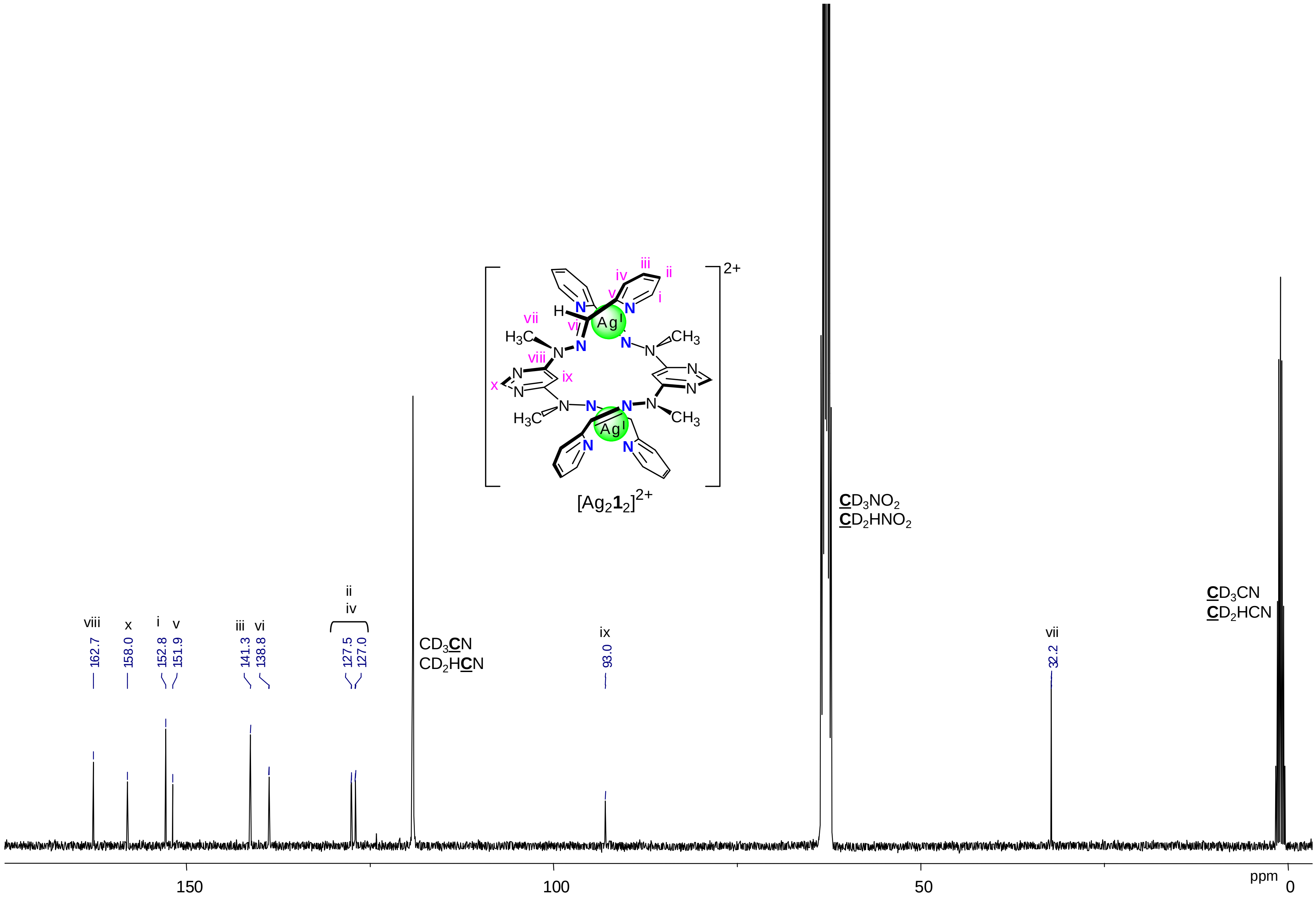

The reaction mixture $Ag_2\mathbf{1}_2(OTf)_2 + Ag_2\mathbf{2}_2(OTf)_2 \rightarrow 2\ Ag_2(\mathbf{1})(\mathbf{2})(OTf)_2$

^{1}H NMR spectrum (400 MHz, $CD_3NO_2/CD_3CN \approx 18/1$ (v/v)) of the reaction mixture $Ag_2\mathbf{1}_2(OTf)_2 + Ag_2\mathbf{2}_2(OTf)_2 \rightarrow 2\ Ag_2(\mathbf{1})(\mathbf{2})(OTf)_2$

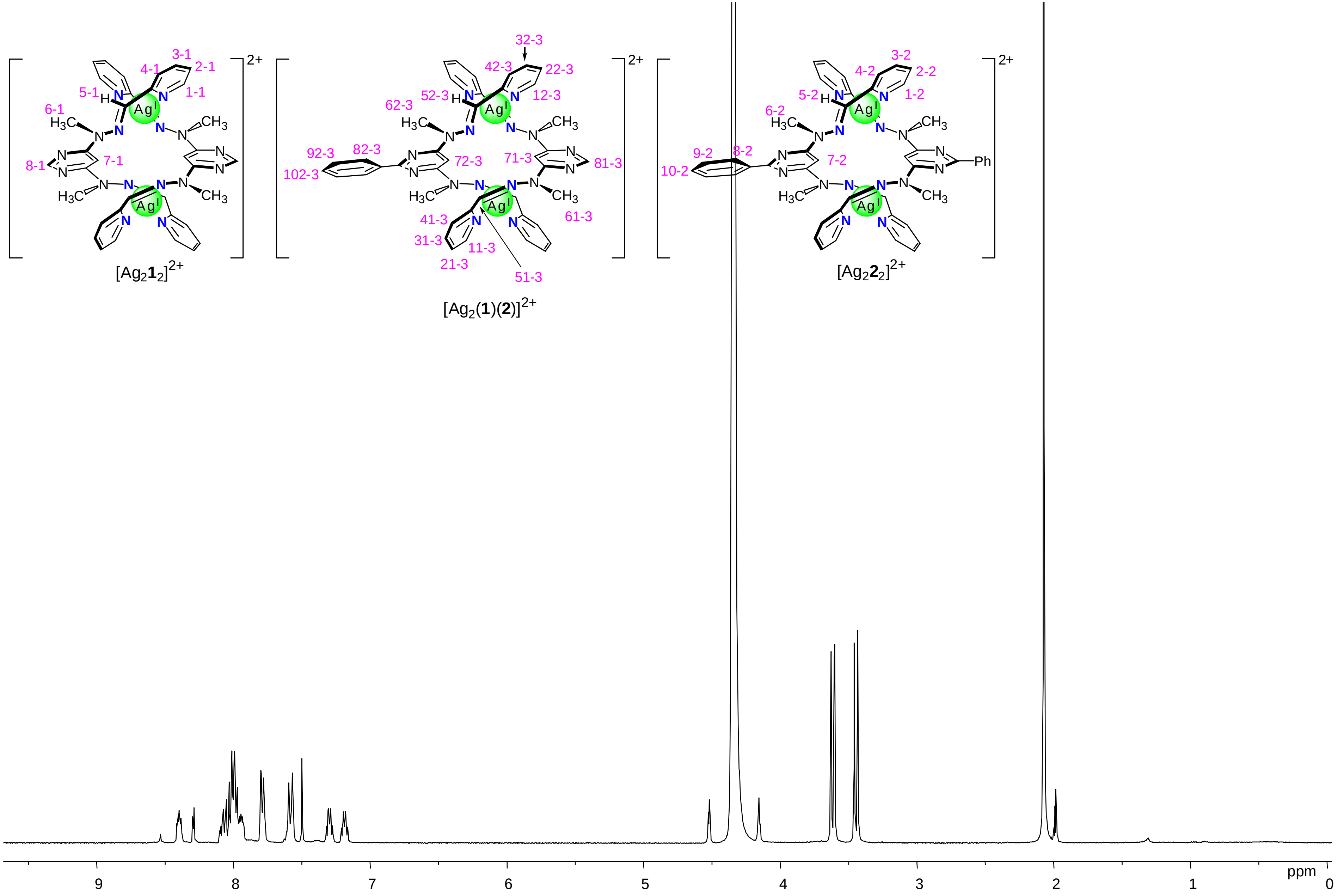

Comparison between the ^{1}H NMR spectra (400 MHz, $CD_3NO_2/CD_3CN \approx$ 16-28/1 (v/v)) of $Ag_21_2(OTf)_2$, $Ag_22_2(OTf)_2$ and of the reaction mixture $Ag_21_2(OTf)_2 + Ag_22_2(OTf)_2 \rightarrow 2\ Ag_2(1)(2)(OTf)_2$

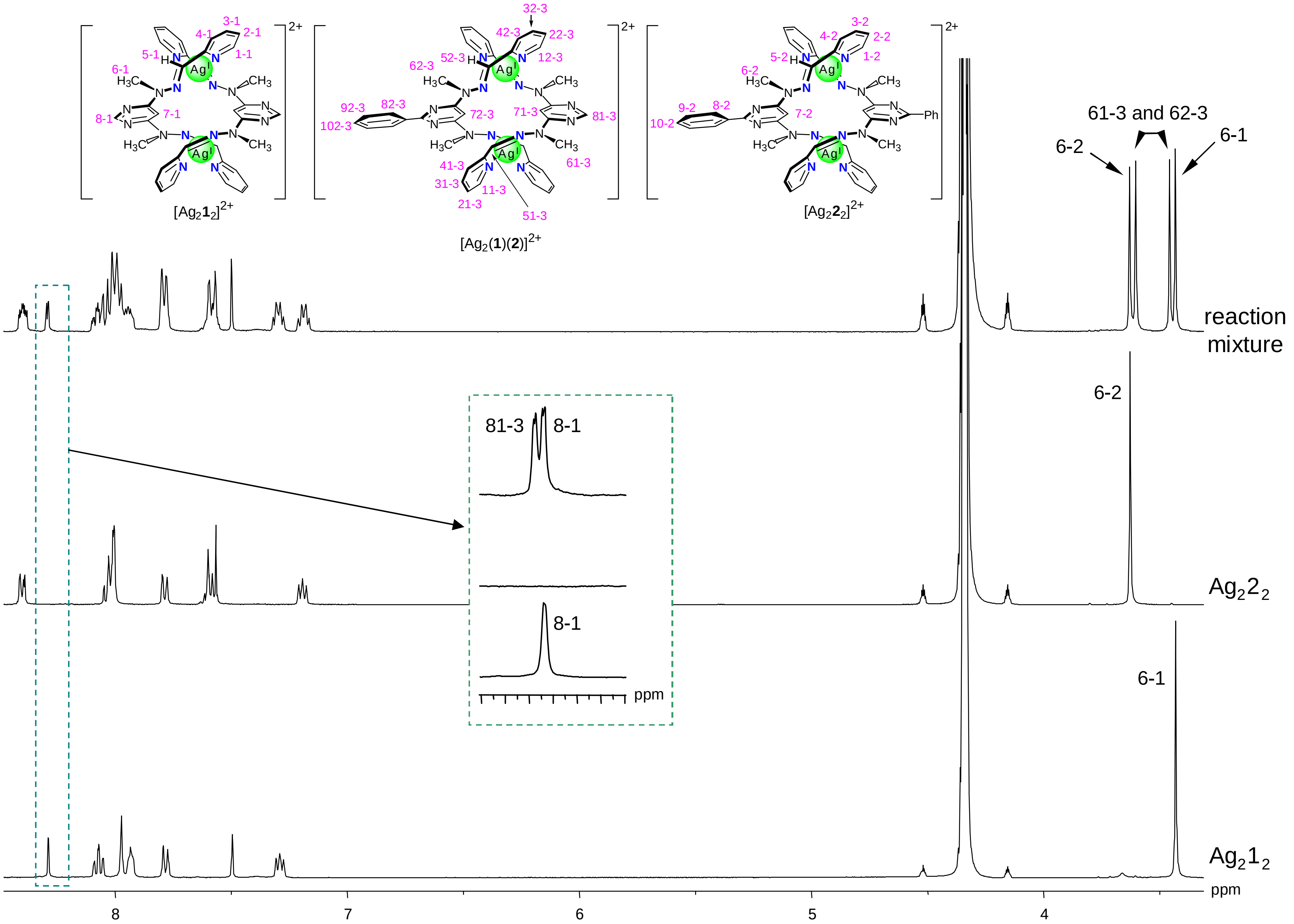

DOSY NMR (600 MHz, $CD_3NO_2/CD_3CN \approx 13/1$ (v/v)) of the reaction mixture $Ag_2 1_2(OTf)_2 + Ag_2 2_2(OTf)_2 \rightarrow 2\ Ag_2(1)(2)(OTf)_2$

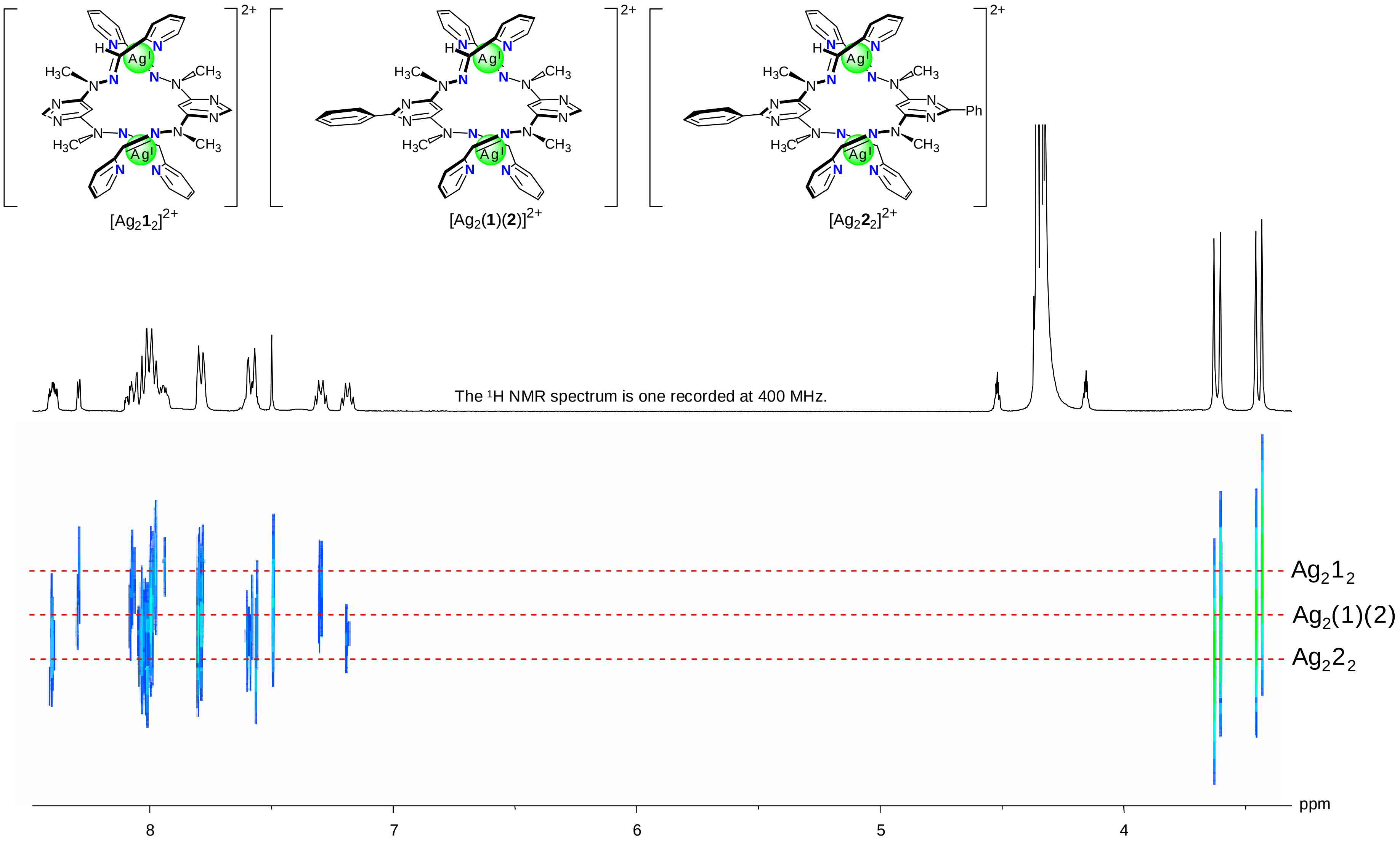

^{13}C NMR spectrum (101 MHz, $CD_3NO_2/CD_3CN \approx 18/1$ (v/v)) of the reaction mixture $Ag_21_2(OTf)_2 + Ag_22_2(OTf)_2 \rightarrow 2\ Ag_2(1)(2)(OTf)_2$

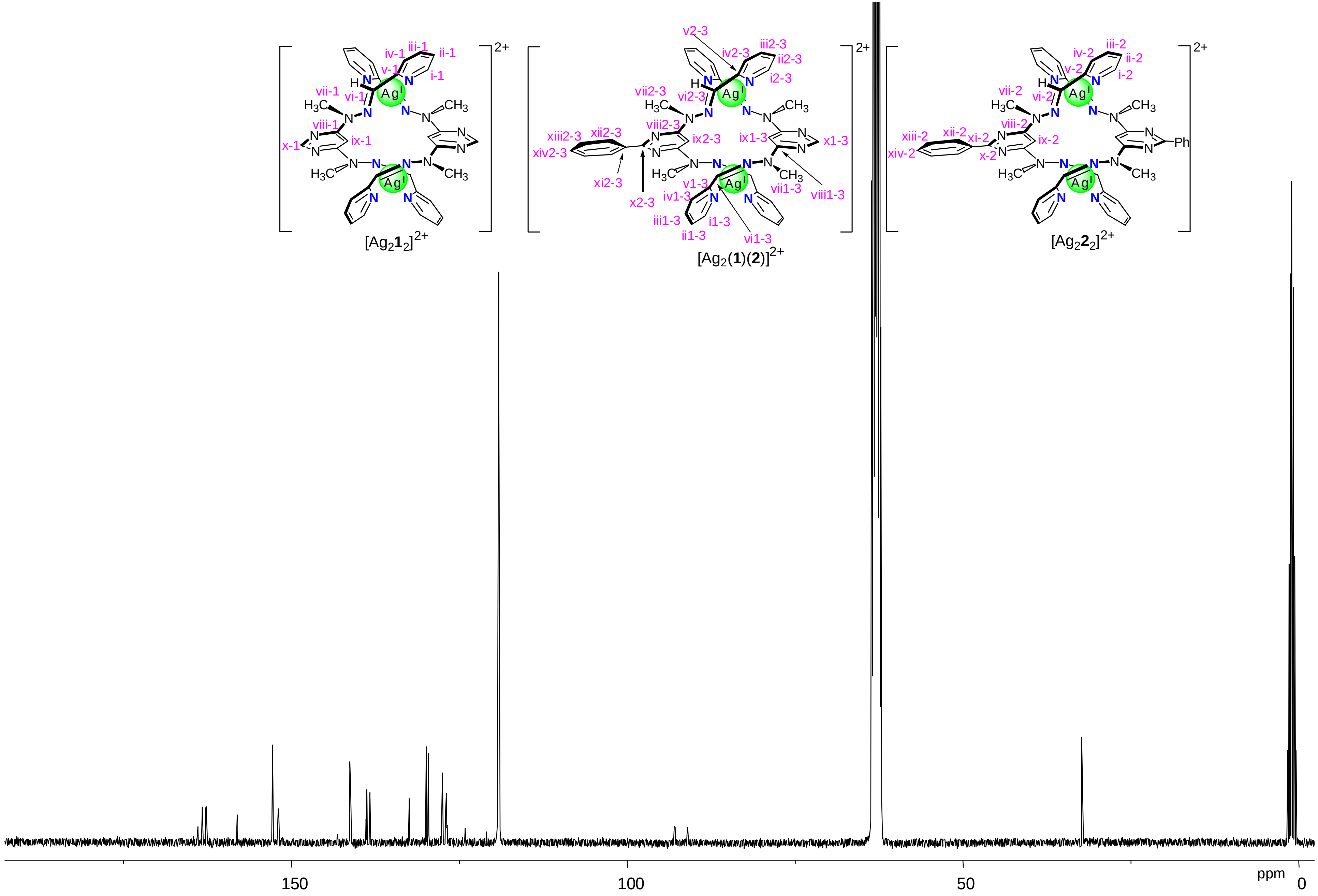

Comparison between the ^{13}C NMR spectra (101 MHz, $CD_3NO_2/CD_3CN \approx$ 16-28/1 (v/v)) of $Ag_2\mathbf{1}_2(OTf)_2$, $Ag_2\mathbf{2}_2(OTf)_2$ and of the reaction mixture $Ag_2\mathbf{1}_2(OTf)_2 + Ag_2\mathbf{2}_2(OTf)_2 \rightarrow 2\ Ag_2(\mathbf{1})(\mathbf{2})(OTf)_2$

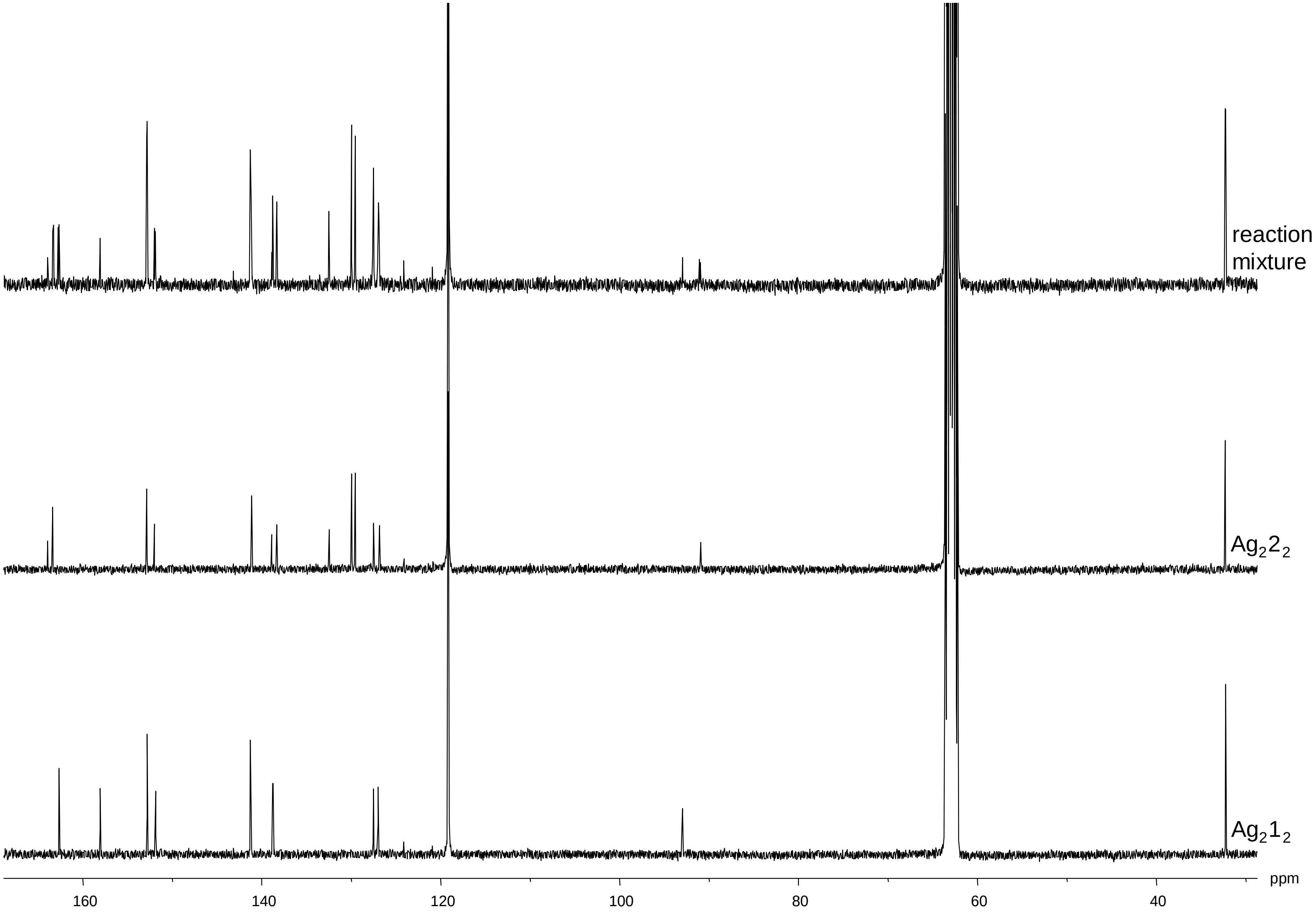

Comparison between the ^{13}C NMR spectra (101 MHz, $CD_3NO_2/CD_3CN \approx$ 16-28/1 (v/v)) of $Ag_21_2(OTf)_2$, $Ag_22_2(OTf)_2$ and of the reaction mixture $Ag_21_2(OTf)_2 + Ag_22_2(OTf)_2 \rightarrow 2\ Ag_2(1)(2)(OTf)_2$

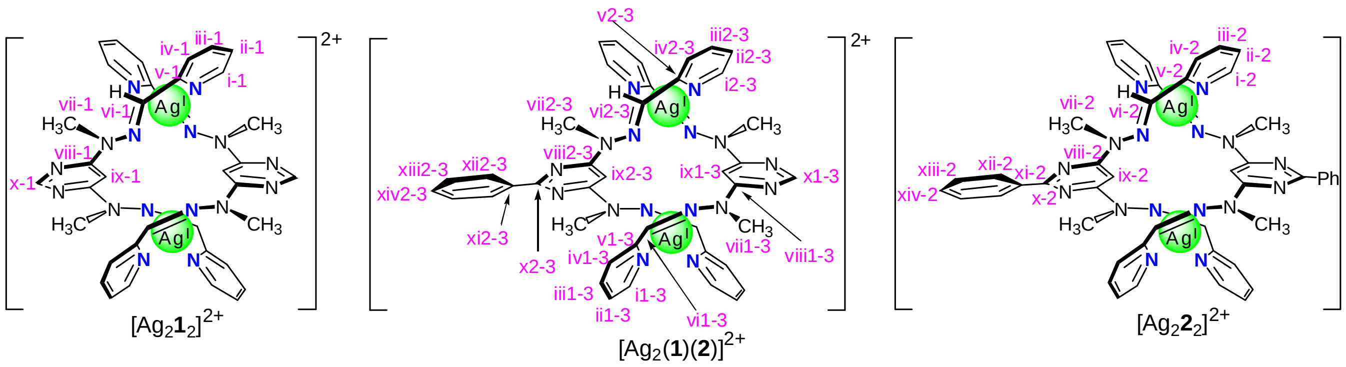

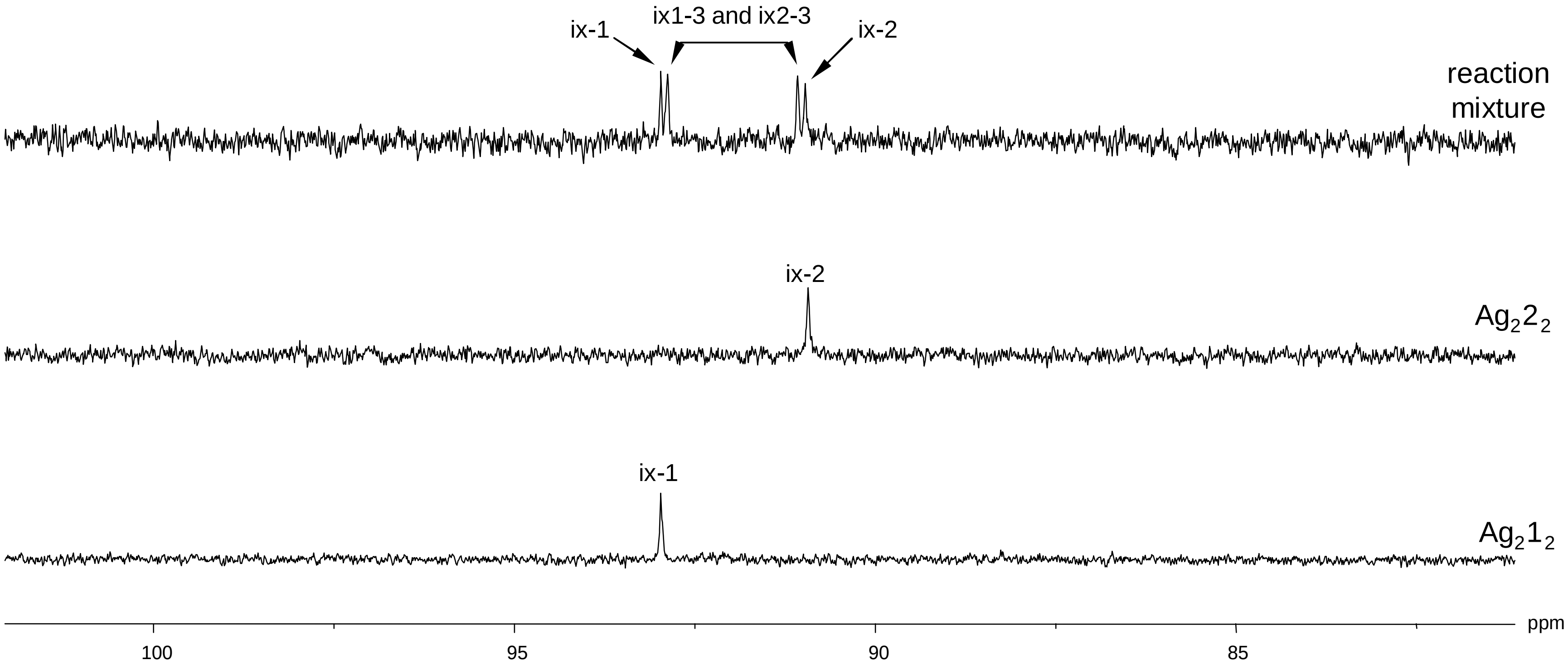

Comparison between the calculated (bottom) and the observed (top) MS peak of $[Ag_2(\mathbf{1})(\mathbf{2})OTf]^+$

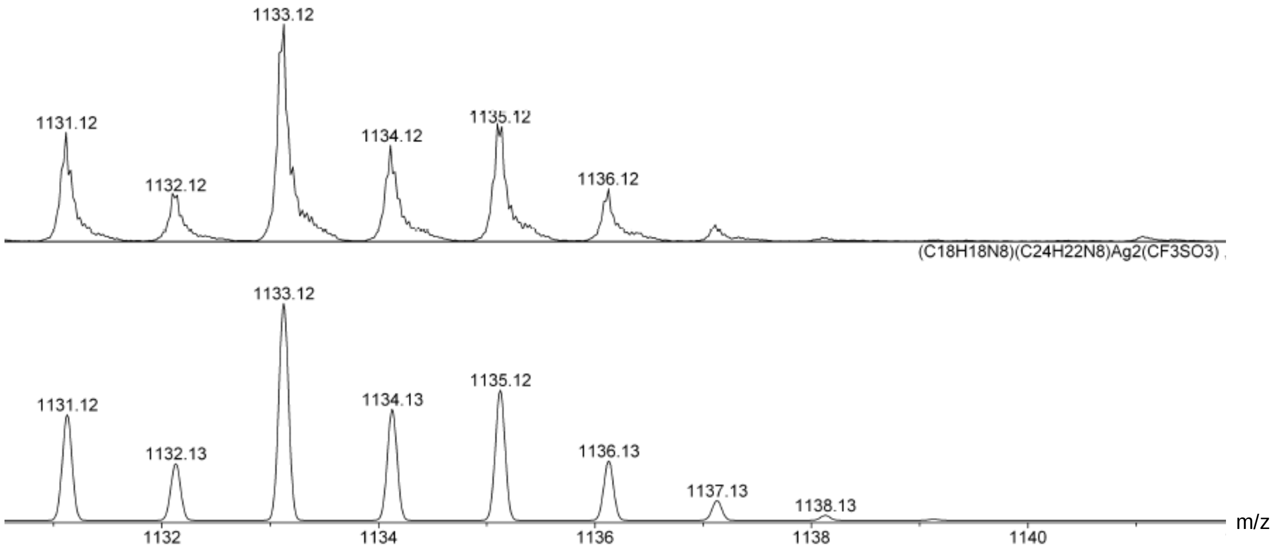

Supplement: Supplementary file 1 [file SC-007-C5SC04403K-s001.pdf]
